# Immune mediated inflammatory diseases: moving from targeted biologic therapy, stem cell therapy to targeted cell therapy

**DOI:** 10.3389/fimmu.2025.1520063

**Published:** 2025-04-07

**Authors:** Zhenguo Liang, Hui Xie, Dongze Wu

**Affiliations:** ^1^ Department of Rheumatology and Immunology, The Fifth Affiliated Hospital, Sun Yat-Sen University, Zhuhai, Guangdong, China; ^2^ Department of Clinical Research and Development, Antengene Corporation, Shanghai, China; ^3^ Department of Rheumatology and Immunology, Sichuan Provincial People’s Hospital, University of Electronic Science and Technology of China, Chengdu, China

**Keywords:** CAR-T, immune mediated inflammatory disease, stem cell therapy, biologic therapy, cell therapy

## Abstract

Despite the advancements in targeted biologic therapy for immune-mediated inflammatory diseases (IMIDs), significant challenges persist, including challenges in drug maintenance, primary and secondary non-responses, and adverse effects. Recent data have strengthened the evidence supporting stem cell therapy as an experimental salvage therapy into a standard treatment option. Recent preclinical and clinical studies suggested that chimeric antigen receptor T cell (CAR-T) therapy, which depleting tissue and bone marrow B cells, may lead to improvement, even inducing long-lasting remissions for patients with IMIDs. In this review, we address the unmet needs of targeted biologic therapy, delineate the critical differences between stem cell transplantation and CAR-T therapy, evaluate the current status of CAR-T therapy for IMIDs and explore its potential and existing limitations.

## 1 Introduction

Immune-mediated inflammatory diseases (IMIDs) comprise both B-cell-mediated autoimmune conditions—including rheumatoid arthritis (RA), systemic lupus erythematosus (SLE), type 1 diabetes (T1DM), pemphigus, and multiple sclerosis (MS)—as well as B-cell-independent chronic inflammatory disorders like psoriasis and other inflammatory diseases (1). The past three decades have witnessed an upsurging new case of IMIDs despite the incidence patterns of IMIDs varied considerably across the world (2). The co-occurrence of 19 IMIDs aligns with shared underlying pathogenetic mechanisms and predisposing factors that interact and vary in influence across these diseases, while also exhibiting distinct dynamic regulatory elements that shape clinical phenotypes and therapeutic responses (3, 4). The advent of targeted biologic therapies, alongside stringent inflammation control, has significantly improved both quality of life and longevity for the clinically diverse spectrum of IMIDs. However, these treatments require continuous administration, rarely restore organ function or reverse disability, and remain non-curative (5). Emerging evidence indicates that stem cell-based therapies, such as mesenchymal stromal cells (MSCs) and hematopoietic stem cell transplantation (HSCT), may achieve sustained, drug-free, and symptom-free remission in multiple refractory IMIDs (6–8).

More importantly, recent data suggest that CD19-targeted chimeric antigen receptor (CAR) T cells therapy brings a ray of healing light to severe refractory IMIDs (9, 10). Beyond HSCT, the adoption of CAR-T cells as a new second-line treatment for patients with refractory or early-relapsing large B-cell lymphoma prompts a reevaluation of CAR-T therapy advancements in IMIDs (11–13).

## 2 Unmet clinical need of targeted biologic therapies for immune mediated inflammatory disease

Current targeted therapies for IMIDs, including biologic agents and small molecule drugs, can achieve sustained low disease activity or remission (14–16). However, unmet needs persist, such as primary and secondary non-response, drug resistance, adverse events, and increased risks of infections, potential malignancies and new onset of another IMIDs. Primary non-response may result from mechanistic failure, while secondary non-response often involves immunogenicity and antidrug antibody (ADAb) formation, which vary across agents (17, 18). ADAbs contribute to therapeutic failure and reduced drug survival rates (19, 20). Furthermore, targeted therapies may trigger new IMIDs or exacerbate coexisting conditions (21). They also heighten infection risks, with serious infections occurring in 3-4% of patients, and may slightly increase malignancy risks, particularly in older patients or with prolonged anti-TNF use (22). Infection, malignancy, risk of VTE, herpes zoster are key points to consider for the treatment of IMID with JAK inhibitors (23, 24).

Although the B cell depletion antibody (BCDA), rituximab, failed to meet primary endpoints in randomized controlled trials for SLE, favorable clinical experience has led to its frequent off-label use. Compared to rituximab, deeper B cell depletion is being explored with antibodies targeting CD20 (obinutuzumab), CD19 (inebilizumab), CD38 (daratumumab), the BAFF receptor (ianalumab), and TACI (telitacicept), as well as proteasome inhibition with bortezomib, all showing promise in IMIDs (25, 26). The SLAMF7-targeting monoclonal antibody elotuzumab, used in multiple myeloma, may also be relevant for certain IMIDs (27). In contrast, monoclonal antibodies targeting CD19 and FcγRIIb (obexelimab) and CD22 (epratuzumab) have not demonstrated efficacy in SLE (28, 29).

A key limitation of BCDA is the increased infection risk due to prolonged B-cell depletion, concurrent immunosuppressive therapy, and hypogammaglobulinemia (30). Additionally, some IMIDs, such as SLE, respond poorly to BCDA, as short-lived autoreactive plasma cells initially susceptible to depletion become resistant once they differentiate into long-lived plasma cells (31, 32). Another possible failure mechanism is the preferential depletion of regulatory B cells or phagocytic defects in macrophages (33). Crucially, the depth of B-cell depletion may determine clinical efficacy in IMIDs. While BCDA effectively depletes circulating B cells, it is less effective in the bone marrow and secondary lymphoid organs, potentially preventing a full ‘reset’ of IMIDs (25).

Despite a plethora of successful targeted biologic therapies with different mechanisms of action, most patients with IMID still do not achieve remission, let alone drug-free remission (34). The durability of biologic treatments remains suboptimal, often necessitating medication switches, which generally result in diminished therapeutic responses. Moreover, biologic drugs have largely failed to cure IMIDs or prevent their onset in most cases (15).

## 3 From stem cell therapy to targeted cell therapy for immune mediated inflammatory diseases

### 3.1 Transition from stem cell therapy to chimeric antigen receptor T cell therapy

**Figure 1:**
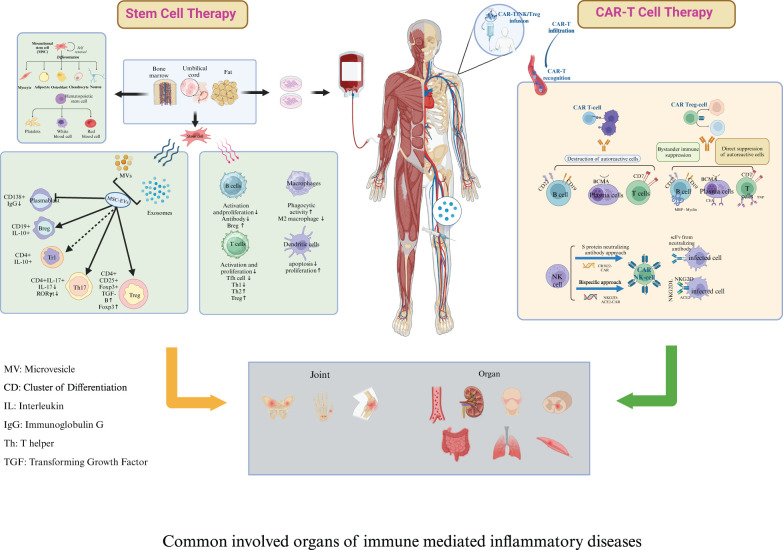
Key differences between stem cell therapy and chimeric antigen receptor T cell therapy. Composition and Structure: Stem cell therapy encompasses both mesenchymal stromal cells (MSCT) and hematopoietic stem cells (HSCT). Both types of stem cells can originate either from the patient (autologous) or a donor (allogeneic). Engineered chimeric antigen receptor (CAR) molecules are hybrid constructs derived from antibodies and T-cell receptors; they include an antigen-binding motif, a hinge domain, a transmembrane domain, one or more costimulatory domains, and a constitutive activation domain (CD3ζ signaling domain). Mechanism: Both stem cell therapy and CAR-T therapy can reboot the immune system and induce *de novo* tolerance in IMIDs. The immunomodulatory and trophic functions of MSCs are likely mediated through the production of soluble factors and the secretion of neurotrophic growth factors. The efficacy of HSCT may stem from the depletion of autoreactive immunologic memory and the generation of a rejuvenated and tolerant immune system. Engineered CAR-T cells specifically target and eliminate pathogenic immune cells, such as autoreactive B cells, thus mitigating the symptoms of such diseases. Furthermore, CAR-T cells exhibit potent depletive effects, enhanced by their superior tissue penetration, intrinsic lethality, and direct cytotoxicity towards plasmablasts and plasma cells. Preparation: MSCT: MSCs are isolated using density gradient centrifugation and adherence methods, cultured, and expanded ex vivo from bone marrow stem cell niches and other sources such as adipose tissue and umbilical cords. HSCT: peripheral blood stem cells are mobilized using cyclophosphamide; peripheral leukocyte counts are monitored, and cells are harvested when the white blood cell level rebounds. CAR-T therapy involves isolating T cells from the patient’s peripheral blood mononuclear cells, engineering these cells to express CARs that recognize specific antigens, expanding the CAR-T cells *in vitro*, and then infusing the engineered cells back into the patient to target and destroy disease-causing cells. Pretreatment: The HSCT pretreatment regimen includes intravenous rabbit antithymocyte globulin and methylprednisolone, administered in a 100-grade laminar flow ward. Cyclophosphamide pretreatment is not recommended before MSCT. Prior to CAR T-cell infusion, patients undergo a conditioning chemotherapy regimen in a standard ward, which includes fludarabine and cyclophosphamide to achieve lymphocyte depletion. Post-infusion Monitoring: For stem cell therapy, post-infusion monitoring encompasses evaluating the success of stem cell implantation, blood cell recovery, occurrence of graft-versus-host disease, and the long-term function and potential complications of the immune system. In the case of CAR-T therapy, post-infusion monitoring focuses on detecting cytokine release syndrome, neurotoxicity syndrome, the persistence and activity of CAR-T cells *In vivo*, and the potential development of secondary malignancies.

In contrast to the chronic immune suppression associated with targeted biologic therapies, stem cell treatments, including both mesenchymal stem cell therapy (MSCT) and hematopoietic stem cell therapy (HSCT)—present a promising approach for potentially curing IMIDs by reinducing self-tolerance. This is supported by evidence from HSCT in MS, SSc, and Crohn’s disease (6), as well as significant symptom improvement in patients with SLE and IBD following MSCT (35). These therapies target the long-term and extensive depletion of immune cells, particularly within the B and T cell populations (6, 36, 37). HSCT offers the advantage of complete eradication of autoreactive cells and broad immune system regeneration, promoting tolerance to autoantigens and potential long-term remission in MS, SSc, and SLE (6). However, its major drawbacks include high toxicity from conditioning regimens, increased infection risk, potential relapses, GVHD risk in allo-HSCT, and relatively high treatment-related mortality (38, 39). MSCT provides potent immunomodulatory and anti-inflammatory effects with no need for chemotherapy, no GVHD risk, and fewer severe side effects than HSCT. However, its variable efficacy and uncertain long-term durability remain key drawbacks (37, 40). While HSCT and MSCT have been valuable in immune modulation, their limitations—toxicity, incomplete immune reset, and variability in efficacy—have paved the way for targeted cell therapy as a more precise, effective, and durable treatment for IMIDs. Unlike stem cell therapy, targeted cell therapy leverages immunoengineering to program immune cells to eliminate pathogenic cells directly within diseased tissues, precisely where they drive systemic autoimmunity (41). The application of immunoengineering has enabled T cells to express a chimeric antigen receptor (CAR) specifically targeting B cell surface antigens, revolutionizing the treatment of hematological malignancies and IMIDs (41, 42). CAR-T cells and T cell engineering to express a chimeric autoantibody receptor (CAAR) have emerged as a potentially curative strategy for autoimmunity, with the goal of achieving profound, short-term depletion of the B-cell compartment (43). CAR-T therapy offers selective depletion of pathogenic cells, deep and sustained B-cell depletion in tissues, high personalization, and the potential for long-term remission, but its main drawback is moderate toxicity, including risks of cytokine release syndrome (CRS) and neurotoxicity. Differences between stem cell therapy and CAR-T cell therapy have been identified in terms of composition, structure, mechanisms, preparation, pretreatment, and post-infusion monitoring (**Figure 1**).

### 3.2 Structure, generation and type CAR based therapy

The evolution of CAR-T cell generations has focused on enhancing signal transduction and immune response. First-generation CAR-T cells contain a CD3ζ domain for activation, while second-generation CAR-T cells add a costimulatory domain (e.g., CD28, ICOS, or 4-1BB) to improve efficacy. Third-generation CAR-T cells further enhance activation by incorporating two costimulatory domains alongside CD3ζ. Fourth-generation CAR-T cells, known as TRUCK CARs, build on second-generation designs by introducing inducible cytokine expression, such as IL-12, to enhance the immune response. Fifth-generation CAR-T cells refine previous designs by integrating the IL-2R beta-chain, which activates the JAK/STAT pathway to optimize antigen-dependent T cell responses (44, 45).

In addition, multiple types of CAR-T cells have been engineered to address the complex immunopathology of hematologic malignancy and IMID. CAR-T cells target specific antigens on pathological cells, while CAAR-T cells recognize the B cell receptor (BCR) on self-reactive B cells to eliminate those involved in autoimmune diseases without requiring HLA or TCR recognition. In contrast, CAR-Treg therapy utilizes regulatory T cells to restore immune balance by suppressing excessive immune responses. CAR-NK cell therapies, with favorable safety and diverse sourcing of NK cells, may offer a cost-effective, “off-the-shelf” alternative that improves accessibility (46).

A key consideration in using CAR-T cells for IMID is determining how long they must persist for therapeutic effects. Unlike hematological malignancy, which may require long-term engraftment, a short burst of CAR-T cells might be enough to trigger an “immune reset” (47). Although the CD28 or 4-1BB co-stimulatory domains in CARs enhance the function T cells activation and anti-cancer activity, the ideal co-stimulatory domain for IMID remains unclear. However, minimizing toxicity will likely be a key consideration in the design of CAR therapies specifically for IMID (48).

### 3.3 Mechanisms and dynamics and of CAR-T cell therapy in IMIDs

T-cell-mediated autoimmunity in IMIDs leads to B-cell activation and autoantibody formation. Complete B-cell depletion may not be effective for refractory IMIDs, as residual autoreactive T cells and tissue-resident T cells could still sustain the disease (49). Engineered CAR-T cells can selectively target autoantigens such as CD19, CD7, and BCMA, eliminating autoreactive B cells, T cells, and plasma cells, to modulate the immune microenvironment and alleviate IMID symptoms (50, 51). In consistent with evidence from tumor studies showing that CAR-T cell residency in the bone marrow is more effective than peripheral persistence for tumor control (52, 53), CAR-T therapies not only deplete circulating B cells but also tissue-resident B cells, particularly in secondary lymphoid tissues, although memory B cells in these tissues may be resistant to depletion (49). However, whether CAR-T cell infiltration into tissues is insufficient or limited to certain subsets of CAR-T cells remains unclear. Furthermore, CAR-T cells derived from SLE patients can induce CD19-dependent activity against autologous primary B cells, accompanied by reduced inflammatory cytokine production (54).

Single-cell profiling and in-depth immunophenotyping in hematologic malignancy have demonstrated that long-lasting CD19-redirected CAR-T cells exhibit cytotoxic characteristics, along with sustained functional activation and proliferation, lasting for more than a decade (55, 56). Dynamic tracking of CAR-T cells in patients with anti-SRP necrotizing myopathy or myasthenia gravis suggests that anti-BCMA CAR-T cells may normalize the immune microenvironment, including the reconstitution of B-cell lineages with sustained reductions in pathogenic autoantibodies, inhibition of proliferative activity in CD8+ CAR-T cells, replacement of T cell subpopulations, and suppression of overactivated immune cells (57, 58). *In vivo* dynamic changes of CAR-T cells post-infusion revealed that the infused CAR-T cells were in a highly metabolically active state, with elevated glycolysis and biosynthetic gene expression. They gradually transitioned from a highly proliferative state to a highly cytotoxic state along a developmental trajectory, reaching a late remission phase characterized by a loss of proliferative activity but sustained cytotoxicity. Eventually, both proliferative and cytotoxic signatures in hematologic malignancy declined during the remission phase (59, 60). Longitudinal tracking of clones in blood and cerebrospinal fluid from five neuromyelitis optica spectrum disorder (NMOSD) patients treated with anti-BCMA CAR-T cell therapy suggests that CD8+ cycling CAR-T cells play a predominant role in autoimmunity, primarily derived from CD8+ effector T cells at baseline prior to manufacturing (61). Longitudinal analysis of peripheral blood mononuclear cells from an Scl70+ SSc patient suggested that CD19-CAR-T cell therapy reverses adaptive autoimmunity and restores Fcγ-receptor IIIA-expressing innate immune cells by reducing immune complex activity (62).

## 4 Preclinical studies of CAR-T therapy for immune mediated inflammatory diseases

The preclinical studies of CAR-T therapy for IMIDs were summarized in **Table 1**.

**Table 1:** Preclinical studies of CAR-T therapy for immune mediated inflammatory diseases.

First author	Year	Recognition domain	Carrier cells	Target cells	Dosage	Persistence
Mouse model of lupus						
Rita Kansal (63)	2021	CD19 scFv	CAR- T	CD19+B cells	1.2*10^7cells	(63)
Xuexiao Jin (64)	2021	CD28 or 4-1BB	CAR- T	CD28+B cells	6.7*10^6cells	
Vinayak Uppin (65)	2024	CD19 scFv	BAFF CAR-T	CD19+B cells	1 × 10^6cells	3\8\12\ 22 weeks
ANCA-induced acute kidney injury						
Dörte Lodka (66)	2024	CD3ζ	CAR- T	SP6 or CD19	2.0*10^6 cells	2\5\8 weeks
Multiple sclerosis						
Moa Fransson (67)	2012	CAR/FoxP3_Tregs	CAR/FoxP3_Tregs	Myelin oligodendrocyte glycoprotein	1.0*10^5cells	7 days
Meike Mitsdoerffer (68)	2021	CD19 scFv	T cell-conditional α4 integrin	CD19+ B cells	1.0–2.0*10^6 cells	4 days
Sasha Gupta (69)	2023	CD19 scFv	CAR- T	CD19+ B cells	2.0*10^6cells	
Autoimmune encephalitis						
S Momsen Reincke (71)	2023	CD8	NMDAR-CAART	B cell receptors (BCRs)	1,0*10^6cells	13 days
Cody D Moorman (72)	2023	anti-human myostatin scFv	CAR-T/CAR-Tregs	XCR1	3 ng/mL/1.0*10 ^6cells	7-21days
Autoimmune cholangitis						
Hao-Xian Zhu (73)	2024	CD28 and CD3ζ moieties	CAR-T	CD8+ T Cells	1.0*10^6 cells	
Graves’ disease						
Honghong Duan (74)	2023	Signal domain (4-1BB)	TSHR-CAR-T	B cells	2.0*10^6 cells	
Type 1 diabetes						
Sigal Fishman (75)	2017	N terminus of β2m/CD3-ζ,	TCR	CD8+ T Cells	6–10*10^6 cells	
Li Zhang (78)	2018	GFP+CD8+ T cells	287-CAR T	CD4+ T cells	5.0*10^6 cells	
Michel Tenspolde (79)	2019	insulin-specific scFvs	CAR-Tregs	Insulin	2.5*10^6 cells	
Ilian A Radichev (77)	2020	Anti-HPi2 scFv	HPi2-CAR	CD98	1.0*10^6 cells	48 hours
Shio Kobayashi (76)	2020	I or II MHC molecules (pMHCI/II)	5MCAR	Antigen-specific T cells		
Justin A Spanier (80)	2023	CD28 and CD3ζ signaling domains.	CAR Tregs	CD4+ T Cells	1.5*10^6 cells	20weeks
Hemophilia A						
Kalpana Parvathaneni (81)	2018	CAR scFv	BAR	CD8+ T cells	1.0*10^6 cells	
Asthma						
Jelena Skuljec (82)	2017	CEA	CAR Tregs	Tregs	5.0*10^4 cells/well	
Gang Jin (84)	2024	5TIF4	IL-5 CAR T cells	BCOR and ZC3H12A	1 mg/kg	5 days a week
Cardiac fibrosis						
Haig Aghajanian (85)	2019	CD3ζ and CD28 cytoplasmic domains	CAR T	MHC I	1.0*10^7 cells	1 week
Joel G Rurik (86)	2022	CD5	CAR T	Lymphocytes		
Myasthenia gravis						
Sangwook Oh (87)	2023	CD137-CD3ze	MuSK-CAART	Anti-MuSK autoantibody or B cell receptor (BCR)	1.0*10^7 cells	
Pemphigus vulgaris						
C M Proby (88)	2000	Terminal EC1 and part of EC2	Dsg3-GFP	B cells	5 ug	12 weeks
Christoph T Ellebrecht (89)	2017	Dsg3 EC1-3/EC1-4/EC1-5	Dsg3 CAAR-T	B cell receptor (BCR)		
Systemic sclerosis						
Jérôme Avouac (90)	2023	CD19 scFv	CAR T	CD19+B cells	10 - 20*10^6 cells	6 weeks
IgG4-related disease						
Yeting Sun (93)	2024	CD28 or 4-1BB	CAR-T	CD19+B cells	1.5 × 10^6 cells	24hours/9 weeks
Ulcerative colitis						
Dan Blat (94)	2014	CD28 and CD3ζ moieties	CAR Tregs	Colorectal tumor	1.0*10^6 cells	

### 4.1 Mouse models of lupus

In two lupus mouse models (NZB/W and MRL-lpr strains), CD19 CAR-T cell transfer induced efficient and stable B cell depletion, elimination of circulating anti-DNA antibodies, and improvements in kidney function, spleen size, lymphocyte subset ratios, and skin inflammation, as well as a significant increase in lifespan (63). Furthermore, in the MRL-lpr lupus model, transfer of syngeneic anti-CD19 CAR-T cells resulted in a more sustained B-cell depletion effect than antibody treatment, prevented disease pathogenesis before the onset of symptoms, and exhibited therapeutic benefits at later stages of disease progression (64). Moreover, multiple preclinical models show that B cell activating factor (BAFF) CAR-T cell therapy effectively depletes autoreactive B cells, offering potential benefits such as avoiding B cell aplasia and preventing long-lived plasma cell escape, though it may also deplete normal mature B cells (65).

### 4.2 ANCA-induced acute kidney injury

In a preclinical model of myeloperoxidase-anti-neutrophil cytoplasmic autoantibody (MPO-ANCA)-associated vasculitis (AAV), CD19-targeted CAR T cells effectively depleted B cells and plasmablasts, promoted a decline in MPO-ANCA levels, and, crucially, provided protection against necrotizing crescentic glomerulonephritis (66).

### 4.3 Multiple sclerosis

The CD4+ T cells were genetically engineered to express a CAR targeting myelin oligodendrocyte glycoprotein (MOG) in combination with the murine FoxP3 gene, resulting in a reduction of disease symptoms and a decrease in IL-12 and IFN-γ mRNA levels in brain tissue from a murine model of MS (67). In a mouse model of MS, treatment with anti-CD19 CAR-T cells resulted in the elimination of meningeal B cell aggregates and exacerbated clinical disease; however, in another B-cell-dependent MS model, anti-CD19 CAR-T cells effectively depleted B cells in both peripheral tissues and the central nervous system (CNS), leading to a reduction in clinical scores and lymphocyte infiltration (68, 69). These contrasting findings in two MS models highlight the diverse autoimmune mechanisms that drive tissue pathology, emphasizing the importance of careful model selection aligned with the primary objectives of the study when evaluating CAR-T therapies (70).

### 4.4 Autoimmune encephalitis

Anti-N-methyl-d-aspartate receptor chimeric autoantibody receptor (NMDAR-CAAR) T cells, which consist of an extracellular multi-subunit NMDAR autoantigen and intracellular 4-1BB/CD3ζ signaling domains, effectively depleted engineered B cells expressing an NMDAR-reactive antibody, resulting in a reduction of serum and brain autoantibodies, with no evidence of off-target toxicity or adverse events (71). X-C motif chemokine receptor 1-specific chimeric antigen receptor T cells (XCR1-CAR-T) and CAR-Tregs led to the depletion of DC1, modestly suppressing the onset of Th1-driven experimental autoimmune encephalomyelitis (72).

### 4.5 Autoimmune cholangitis

Primary biliary cholangitis is characterized by an autoreactive T cell response targeting intrahepatic small bile ducts. CD8+ tissue-resident memory T cells induce apoptosis of bile duct epithelial cells and upregulate PD-1 expression. Treatment of DKO mice with PD-1 CAR-T cells selectively depleted liver CD8+ tissue-resident memory T cells and alleviated autoimmune cholangitis (73).

### 4.6 Graves’ disease

Graves’ disease (GD) is caused by stimulating antibodies that target the thyroid-stimulating hormone receptor (TSHR), referred to as TSH receptor antibodies (TRAb). TSHR-CAR-T was constructed by incorporating the first extracellular domain (21-413aa) of TSHR, fused with the CD8 transmembrane and intracellular signaling domain (4-1BB), which can recognize and effectively eliminate TRAb-producing B lymphocytes both *in vitro* and *in vivo (* 74).

### 4.7 Type 1 diabetes

First-generation CD3-ζ-CAR T cells, expressing the β2-microglobulin component of MHC I and targeting the anti-InsB15-23-H-2Kd TCR, significantly reduced insulitis and protected non-obese diabetic (NOD) mice from T1DM (75). Another first-generation CAR cytotoxic T lymphocytes, equipped with five modules targeting clonotypic TCRs expressed by CD4+ T cells, could eliminate pathogenic CD4+ T cells, neutralize their impact on T1DM, and reduce insulitis in the pancreas of treated NOD mice (76). Two types of CD28-CAR Tregs targeting the human pancreatic endocrine marker Hpi2 demonstrated no immunologic function against the Hpi2 antigen *in vitro*, due to the broad expression of the Hpi2 antigen across multiple cell types. This underscores the importance of careful selection of the CAR recognition target (77). None of the 287 NOD mice treated with second-generation CD28-CAR and 4-1BB-CAR CD8+ T cells targeting the I-Ag7-B:9-23(R3) complex developed T1DM before 18 weeks of age; however, protection declined over time, indicating that these CAR-T cells can only delay, but not prevent, the development of T1D (78). The second-generation CD28-CAR Tregs targeting soluble insulin were unable to prevent T1DM development in NOD mice but remained present even after four months (79). Another second-generation CD28-CAR Tregs targeting the insulin B-chain 10-23 peptide MHC complex were constructed and co-transferred, preventing adoptive transfer diabetes induced by BDC2.5 T cells in immunodeficient NOD mice. Additionally, these Tregs prevented spontaneous diabetes in WT NOD mice (80). The use of InsB-g7 CAR Tregs for the reversal of autoimmune diabetes following islet transplantation may be more amenable to clinical translation than disease prevention.

### 4.8 Hemophilia A

As 20% to 30% of patients receiving factor VIII (FVIII) gene therapy (F8) produce neutralizing anti-FVIII antibodies, an engineered B-cell antibody receptor (BAR) targeting the immunodominant FVIII domains (A2 and C2) was developed. This receptor could effectively eliminate FVIII-reactive B-cell hybridomas both *in vitro* and *In vivo*, significantly reducing anti-FVIII antibody formation in hemophilic mice (81).

### 4.9 Asthma

Allergic airway inflammation is characterized by airway hyper-reactivity (AHR) and a chronic, T helper-2 (Th2) cell-dominated immune response to allergens. Carcinoembryonic antigen (CEA), which is expressed on the surface of adenoepitheliomas in the lung and gastrointestinal tract, was targeted by the development of an anti-CEA chimeric antigen receptor (CAR). Subsequently, CD4+CD25+ regulatory T cells (Tregs) and CD4+CD25− effector T cells (Teffs) expressing the anti-CEA CAR were isolated from the spleens of anti-CEA CAR-transgenic (CARtg) mice (82). The targeted CD4+CD25+ regulatory T cells (Tregs) significantly reduced airway hyper-reactivity, airway eosinophilia, mucus hypersecretion, Th2 cytokine production, and allergen-specific IgE after sensitization with a model allergen. Furthermore, they efficiently suppressed the proliferation of CAR-expressing effector T cells (Teffs) *in vitro*, either upon stimulation through their T cell receptor (TCR) or via CAR binding to the cognate antigen (82). Given that IgE is a key mediator of allergic responses, IgE-expressing cells can be targeted by T cells through the recognition of the transmembrane form of IgE (mIgE), via the extracellular domain of the high-affinity IgE receptor, FϵRIα. Low-affinity FcϵRIα-based CARs are capable of mediating potent and specific T cell responses against mIgE-expressing target cells, even in the presence of secreted IgE (83). Type 2-high asthma, characterized by IL-5-driven eosinophilia, prompted the development of long-lived CAR T cells engineered with IL-5 as the targeting domain. These cells deplete BCOR and ZC3H12A, leading to sustained repression of lung inflammation and alleviation of asthmatic symptoms in asthma models (84).

### 4.10 Cardiac fibrosis

Excessive cardiac fibrosis is associated with various forms of cardiac disease and heart failure; however, limited clinical interventions are available to address cardiac fibrosis. Adoptive transfer of T cells expressing CARs targeting fibroblast activation protein (FAP), an endogenous marker of cardiac fibroblasts, results in a significant reduction in cardiac fibrosis and the restoration of function after injury in mice (85). Transient antifibrotic CAR-T cells were generated *In vivo* by delivering modified messenger RNA (mRNA) encapsulated in T cell–targeted lipid nanoparticles (LNPs). Treatment with modified mRNA-targeted LNPs reduced fibrosis and restored cardiac function after injury, highlighting the promising potential of *In vivo* generation of CAR T cells as a therapeutic platform for treating various diseases (86).

### 4.11 Myasthenia gravis

T cells were genetically engineered to express a MuSK chimeric autoantibody receptor (MuSK-CAART) containing CD137-CD3ζ signaling domains, which demonstrated comparable efficacy to anti-CD19 chimeric antigen receptor T cells in depleting anti-MuSK B cells and retained cytolytic activity even in the presence of soluble anti-MuSK antibodies (87).

### 4.12 Pemphigus vulgaris

A chimeric autoantigen-toxin molecule, consisting of the immunoreactive portion of the extracellular domain of desmoglein 3 and Pseudomonas exotoxin, was constructed to specifically recognize and eliminate autoimmune B cells in pemphigus vulgaris (PV). The chimeric molecules have been shown to bind and partially kill B cell hybridomas producing anti-desmoglein 3 monoclonal antibodies. They can also partially eliminate antigen-specific B cells from the spleens of mice immunized with desmoglein 3 (88). A CAAR, consisting of the PV autoantigen desmoglein (Dsg) 3 fused to CD137-CD3z signaling domains, exhibited specific cytotoxicity against cells expressing anti-Dsg3 BCRs *in vitro*. It also expanded, persisted, and specifically eliminated Dsg3-specific B cells *In vivo* (89). DSG3 CAAR-T cells selectively kill DSG3-reactive B cells while sparing non-affected B cells, and they do not affect keratinocytes either *in vitro* or *in vivo.*

### 4.13 Systemic sclerosis

In the Fra-2 transgenic (Tg) mouse model, depleting circulating B cells with anti-CD20 mAb infusion three days before CAR-T cell injection resulted in deeper B-cell depletion in both peripheral blood and lesional lungs compared to anti-CD20 mAb alone. However, CAR-T cell infusion significantly increased lung collagen content, histological fibrosis scores, and right ventricular systolic pressure, while also worsening clinical outcomes and increasing mortality in Fra-2 Tg mice (90). While anti-CD20 mAb pre-treatment may enhance CAR-T cell engraftment and persistence (91), it could also lead to unpredictable adverse effects. The persistence and accumulation of CD19-targeted CAR-T cells in lesional lung tissue may be exacerbated by severe lung inflammation. Given the extensive lung involvement in the Fra-2 model compared to murine lupus (92), these findings suggest that CD19-targeted CAR-T cells may not be suitable for initial use in SSc patients with a high lung inflammatory burden.

### 4.14 IgG4-related disease

Dysregulated B-cell activation drives IgG4-related disease (IgG4-RD). In a LatY136F knock-in mouse model, CD19-targeted CAR-T cell infusion depleted B-cells and reduced CD138+ plasma cells, with CD28 co-stimulatory domain CAR-T cells showing greater efficacy than 4-1BB CAR-T cells. Importantly, CAR-T cells derived from IgG4-RD patients demonstrated effective *in vitro* functionality, highlighting their potential for clinical application (93).

### 4.15 Ulcerative colitis

Given that carcinoembryonic antigen (CEA) is overexpressed in both human colitis and colorectal cancer, CEA-specific CAR regulatory T cells (Tregs) were developed. These cells suppress the severity of colitis in the T-cell-transfer colitis model and reduce the subsequent colorectal tumor burden in the azoxymethane–dextran sodium sulfate model for colitis-associated colorectal cancer (94).

## 5 Clinical studies and cases of CAR-T therapy for immune mediated inflammatory diseases

The clinical cases of CAR-T therapy for IMIDs were summarized in **Table 2**. Generally, CAR-T cells have exhibited significantly longer persistence in clinical cases than preclinical studies (**Tables 1**, **2**).

**Table 2:** Clinical studies and cases of CAR-T therapy for immune mediated inflammatory diseases.

First author	Year	Recognition domain	Carrier cells	Target cells	Dosage	Persistence
SLE with lymphoma						
Jacob L Schmelz (96)	2019	CD19 scFv	CAR- T	CD19+B cells		30 days
Wenli Zhang (97)	2021	CD19-BCMA	CAR- T	BCMA-CD19	5.3*10^6/kg	2 months
Jiasheng Wang (98)	2023	CD19 scFv	CAR- T	CD19+B cells		6 months
Case series of SLE						
Dimitrios Mougiakakos (99)	2021	CD19 scFv	CAR- T	CD19+B cells		
Andreas Mackensen (9)	2022	CD19 scFv	CAR- T	CD19+B cells	1.0*10^6/kg	10 days
Afroditi Boulougoura (101)	2023	CD19 scFv	CAR- T	CD19+B cells	1.0*10^6/kg	3 months
Xue He (107)	2025	CD19 scFv	CAR-T	CD19+B cells	1 × 10^5	60 days
Melanie Hagen (109)	2024	CD19 scFv and 4-1BB	CAR-T	CD19+B cells	1.0*10^6/kg	12 weeks
Daniel Nunez (100)	2023	CD19 scFv and 4-1BB	CAR-T	CD19+B cells	1 × 10^8	3 months
Tobias Krickau (105)	2024	CD19	CAR- T	CD19+B cells	1.0*10^6/kg	
Weijia Wang (108)	2024	CD3ζ, CD28 and 41BB	cCAR-T	BCMA and CD19+B cells	3.0*106/kg	3 months
Mengtao Li (102)	2024	CD19	CAR- T	CD19+B cells	5*10^3/kg	14days
Josefina Cortés Hernández (103)	2023	CD19 scFv	CAR T	CD19+B cells	12.5*10^6	28 days
J. Cortés-Hernández (104)	2024	CD19 scFv	CAR T	CD19+B cells	12.5*10^6	
C. Bracaglia (106)	2024	CD19 scFv	CAR T	CD19+B cells	1.0*10^6/kg	3 months
Lymphoma concurrent with anti-phospholipid syndrome						
Eleonora Friedberg (114)	2025	CD19 scFv	CAR T	CD19+B cells		1 year
Jacob L Schmelz (96)	2020	CD19 scFv	CAR T	CD19+B cells		30 days
Idiopathic inflammatory myopathy						
Fabian Müller (115)	2023	CD19 scFv	CAR- T	CD19+B cells	1.0*10^6/kg	
Ann-Christin Pecher (116)	2023	CD19 scFv	CAR- T	CD19+B cells	1.23*10^6/kg	
Jule Taubmann (117)	2024	CD19 scFv	CAR- T	CD19+B cells	1.0*10^6/kg	
Chuan Qin (61)	2024	CD19 scFv	CAR-T	CD19+ CD27− IgD+ naive B cells	1.0*10^6	18 months
Fabian Müller (10)	2024	CD19 scFv	CAR- T	CD19+B cells		
Rebecca Nicola (118)	2024	CD19 scFv	CAR- T	CD19+B cell	1× 106 CAR T cells/kg	8 months
Xiaobing Wang (120)	2024	CD19 scFv	CAR- T	CD19+B cells	1.0*10^6/kg	3 months
Jenell Volkov (119)	2024	CD19 scFv	CAR T	CD19+B cells	1.0*10^6/kg	4 months
Systemic sclerosis						
Christina Bergmann (121)	2023	CD19 scFv	CAR- T	CD19+B cells	1.0*10^6/kg	
Wolfgang Merkt (122)	2024	CD3ζ, CD28 and 41BB	CAR- T	CD19+B cells	5.0*10^6/kg	
Fabian Müller (10)	2024	CD19 scFv	CAR- T	CD19+B cells		
Janina Auth (124)	2025	CD19 scFv	CAR T	CD19+B cells	1.0*10^6/kg	3, 6, 9, and 12 months
Rheumatoid arthritis						
Aiden Haghiki (126)	2024	CD19 scFv	CAR- T	CD19+B cells	1×10^8	5 months
Merav Lidar (129)	2025	CD19 scFv	CAR T	CD19+B cells	1.0*10^6/kg	100 days
Aliya Masihuddin (128)	2024	CD19 scFv	CAR-T	CD19+B cells		
Fredrik N Albach (127)	2025	CD19 scFv	CAR-T	CD19+B cells		56 days
Yujing Li (130)	2025	anti-IL-6 single chain variable fragments (scFv) and anti-TNFα scFv	CAR-T	CD19/aIL-6/aTNFα		
Aiden Haghiki (126)	2024	CD19 scFv	CAR- T	CD19+B cells	1×10^8	5 months
Pemphigus vulgaris						
Felix Fischbach (132)	2024	CD19	CAR- T	CD19+B cells	1.0*10^8	
Multiple sclerosis						
Felix Fischbach (132)	2024	CD19 scFv	CAR T	CD19+B cells	1.0*10^8	3 months
Neuromyelitis optica spectrum disorders						
Chuan Qin (134)	2023	BCMA scFv	CAR- T	B-cell maturation antigen (BCMA)	1.0*10^6/kg	2 years
Myasthenia gravis						
Volkan Granit (135)	2023	plasma cells	rCAR-T	B-cell maturation antigen (BCMA)	3.5*10^6/kg	
Aiden Haghikia (136)	2023	CD19	CAR-T	B cells and autoantibodies	1.0*10^8	62 days
Dai-Shi Tian (58)	2024	CD8	CAR-T	B cells	1.0*10^6/kg	18 months
Jeremias Motte (137)	2024	CD19 scFv	CAR-T	B cells	1 × 10^8	154 days
Stiff-person syndrome						
Simon Faissner (138)	2024	CD19 scFv	CAR T	CD19+B cells	1 × 10^8	3 months
ANCA-associated vasculitis						
Ioanna Minopoulou (139)	2025	CD19 scFv	CAR T	CD19+B cells	1.0*10^6	6 weeks

### 5.1 Lymphoma concurrent with SLE

CAR-T therapy for lymphoma has shown dual benefits, improving both lymphoma and concurrent SLE or anti-phospholipid syndrome (95, 96). Two studies have demonstrated the therapeutic potential of CD19 CAR-T cell therapy in patients with concurrent SLE and lymphoma. A 41-year-old female with a 20-year history of SLE, recently diagnosed with stage IV diffuse large B-cell lymphoma, who received BCMA-CD19 compound CAR-T cells, exhibited reductions in nuclear, cytoplasmic, and granular ANA and total immunoglobulin levels, achieving remission of both SLE and lymphoma (97). Furthermore, fifty-eight patients with concurrent B-cell non-Hodgkin lymphoma and IMIDs who received CD19 CAR-T cell therapy showed significant reductions in inflammatory markers, seronegative conversion of autoantibodies, and a decrease in the use of steroids and disease-modifying anti-rheumatic drugs (98).

### 5.2 Case series of SLE

A case series with follow-up of eight SLE patients has raised cautious optimism for anti-CD19 CAR-T cell therapy in SLE, particularly regarding deep B-cell depletion, disease remission, seroconversion, cytokine modulation, refractory immune thrombocytopenia, reactivity profiles, GMP-grade preparation, and safety (9, 10, 99–104). In contrast to adult SLE, anti-CD19 CAR-T cell therapy also rescued pediatric SLE patients with/without lupus nephritis (LN) (105–107). More importantly, a Phase 1 study of BCMA and CD19-directed compound CAR-T therapy demonstrated the ability to simultaneously reset the humoral and B-cell immune systems and deplete disease-causing autoantibodies derived from B cells and long-lived plasma cells in patients with SLE/LN (108). CNS involvement, including encephalitis and transverse myelitis, is a severe manifestation of SLE. Notably, a 21-year-old man with SLE and progressive transverse myelitis showed improved neurological function, muscular strength, and regression of MRI lesions after treatment with a second-generation CAR T-cell therapy (CD19 binder and 4-1BB co-stimulation), along with decreased CSF protein (109). Increasing evidence indicates that plasmacytoid dendritic cells (pDCs) play a significant role in the development of IMIDs, primarily through the overproduction of proinflammatory cytokines (110). Two Phase 2 trials have suggested that the elimination of pDCs is beneficial in improving both systemic and cutaneous lupus erythematosus (111, 112). A preliminary study demonstrated the feasibility of producing CD123-targeted CAR-T cells from the T cells of patients with IMIDs, as well as their *in vitro* cytotoxicity against circulating autologous pDCs (113).

### 5.3 Lymphoma concurrent with anti-phospholipid syndrome

The current treatment for antiphospholipid syndrome (APS) involves indefinite anticoagulation with vitamin K antagonists to prevent thrombotic recurrence, with no curative option to permanently eradicate antiphospholipid antibodies (aPL). However, a patient with relapsed or refractory diffuse large B-cell lymphoma (DLBCL) and a nearly 30-year history of APS achieved DLBCL remission, as assessed by PET-CT, and normalization of anticardiolipin antibodies at 1-year follow-up after CAR T-cell treatment (96). Another 65-year-old woman with SLE, APS (triple aPL positivity), and DLBCL achieved ongoing complete remission of DLBCL after anti-CD19 CAR T-cell treatment, along with seroconversion of lupus anticoagulant, anti-cardiolipin, and anti-beta2 glycoprotein I antibodies (114). Both cases suggest a promising role for targeted cell therapy in treating pro-thrombotic autoimmune disorders.

### 5.4 Idiopathic inflammatory myopathy

Several adult cases of CD19 CAR-T cell therapy in Anti-synthetase syndrome have demonstrated resolution of muscle, lung, and skin inflammation, despite prior failure of two B-cell-depleting antibodies, suggesting that the depth of B-cell depletion is significantly greater with CAR-T cell-based therapy than with antibody-based B-cell depletion (10, 115–117). A satisfactory response to anti-CD19 CAR-T cell therapy has recently been reported in a 12-year-old caucasian boy with refractory juvenile dermatomyositis, which was resistant to multiple lines of immunosuppressive treatment, including B-cell depletion with rituximab (118).

There are no FDA-approved therapies for the immune-mediated necrotizing myopathy (IMNM) subtype of idiopathic inflammatory myopathy. The first IMNM patient in the RESET-Myositis™ phase I/II trial (NCT06154252) treated with fully human 4-1BBz anti-CD19 CAR T-cell therapy (CABA-201) showed decreased CK levels, improved muscular strength, depleted peripheral B-cells, and a reduction in autoantibodies to baseline (119). Additionally, a patient with anti-signal recognition particle IMNM, refractory to multiple available therapies, who was treated with BCMA-CAR-T cells, demonstrated a favorable safety profile, sustained reduction in pathogenic autoantibodies, and persistent clinical improvements over 18 months (57). Encouragingly, treatment with genetically engineered, healthy donor-derived CD19-targeted CAR-T cells (TyU19) alleviated severe skeletal muscle damage in a patient with refractory IMNM and reversed extensive fibrotic damage to critical organs in two patients with diffuse cutaneous systemic sclerosis (120).

### 5.5 Systemic sclerosis

The first promising experience with CD19-targeted CAR-T cells in diffuse cutaneous systemic sclerosis (SSc) was reported in a 60-year-old man, who exhibited rapid improvement in heart function, as assessed by 68Ga-FAPI-04 PET-CT, joint health from MRI, and skin fibrosis, alongside seroconversion of antinuclear antibody (ANA) and anti-RNA polymerase III autoantibodies, as well as stable lung function parameters (121). A 38-year-old woman with Scl70+ systemic sclerosis (SSc) and rapidly progressive non-specific interstitial pneumonia, treated with third-generation CD19 CAR-T cells, showed sustained amelioration of lung function and dramatic regression of imaging findings, including the disappearance of Fcγ-receptor-activating immune complexes (122). Four additional patients with SSc, treated with CD19 CAR-T therapy, did not exhibit disappearance of anti-Scl70 antibodies, and their EUSTAR activity index did not regress to negative levels (10). The absence of anti-Scl70 antibody seroconversion, along with reduced EUSTAR activity in all affected organs, suggests that prolonged CAR-T cell persistence may promote seroconversion, potentially leading to extended B-cell depletion, impaired vaccination responses, and an immunocompromised state (123). Therefore, the benefit-risk balance of pretreatment, CAR-T cell persistence and the duration of B-cell depletion must be carefully evaluated. Encouragingly, the same research group recently confirmed that CD19-targeting CAR T-cell therapy may prevent the progression of fibrotic and vascular organ manifestations in SSc patients (124).

### 5.6 Rheumatoid arthritis

Universal anti-fluorescein isothiocyanate (FITC) CAR-T cells, in combination with FITC-labelled citrullinated peptides, have been shown to specifically redirect and eliminate hybridoma cells and autoreactive B cell subsets from RA patients by specifically recognizing FITC-labelled citrullinated peptide epitopes. The cytotoxicity of these CAR-T cells was strictly dependent on the presence of the peptides in a dose-dependent manner (125). Several cases of RA with coexisting myasthenia gravis, SSc, Sjogren’s syndrome, and DLBCL demonstrated that CD19-directed CAR T-cell therapy not only achieved stable clinical remission but also led to seroconversion of anticitrullinated protein antibody (ACPA), indicating the eradication of ACPA-specific plasma cells (126–129). Notably, in three patients with difficult-to-treat RA, fourth-generation CD19-targeted CAR T-cells (CD19/aIL-6/aTNFα) improved symptoms, reduced effusion, synovitis, osteitis, and tendinitis (via ultrasound and MRI), and induced seroconversion of rheumatoid factor and anti-CCP antibodies (130). This study presents a novel therapeutic approach that combines TNF and IL-6 inhibition with B-cell depletion in a single CAR T-cell platform, enhancing RA treatment efficacy while also mitigating CRS.

### 5.7 Pemphigus vulgaris

A first-in-human trial provided preliminary evidence of the efficacy of DSG3-CAART in patients with active anti-DSG3 mucosal PV. This therapy specifically lyses human anti-DSG3 B cells, decreases target cell burden, reduces serum and tissue-bound autoantibodies, and enhances DSG3-CAART engraftment (131).

### 5.8 Multiple sclerosis

Two patients with progressive MS who received CD19 CAR-T cell therapy (KYV-101) showed CAR-T cell presence and expansion in the cerebrospinal fluid, without neurotoxicity, alongside a reduction in intrathecal antibodies in one patient (132). The FDA has granted Fast Track Designation to the autologous, fully human CD19 CAR-T cell product candidate, KYV-101, for the treatment of patients with refractory progressive MS (133).

### 5.9 Neuromyelitis optica spectrum disorders

A first-in-human clinical study of CT103A indicated that BCMA CAR T-cell therapy resulted in improvements in disability and quality of life outcomes, a reduction in AQP-4 antibodies, and a manageable safety profile in patients with relapsed or refractory AQP4-IgG seropositive neuromyelitis NMOSD (134).

### 5.10 Myasthenia gravis

In contrast to the conventional DNA-based approach, autologous BCMA RNA chimeric antigen receptor T cells (BCMA rCAR-T), as exemplified in the Descartes-08 trial, resulted in clinically meaningful reductions in all measures of myasthenia gravis severity, including the induction of minimal symptom expression and the elimination of dependence on intravenous immunoglobulin infusions in adults with generalized myasthenia gravis (NCT04146051) (135). A 33-year-old woman with anti-acetylcholine receptor (anti-AChR)-positive generalized myasthenia gravis received a second-generation anti-CD19 CAR T construct (KYV-101), resulting in a 70% reduction in pathogenic anti-AChR antibodies and the maintenance of protective vaccination IgG titers, which paralleled an improvement in the patient’s muscle strength and reduction in fatigue (136).

Two patients with highly relapsed and refractory myasthenia gravis—one with AChR-IgG and the other with MuSK-IgG—treated with BCMA CAR-T, resulting in B cell lineage reconstitution, exhibited favorable safety profiles and sustained clinical improvements (58). The safe administration of anti-CD19 CAR T cells in two women with concomitant myasthenia gravis and Lambert-Eaton myasthenic syndrome resulted in profound B cell depletion and normalization of acetylcholine receptor and voltage-gated calcium channel N-type autoantibody levels, which paralleled significant neurological responses (137).

### 5.11 Stiff-person syndrome

A 69-year-old female with a 9-year history of treatment-refractory stiff-person syndrome, characterized by deteriorating episodes of stiffness, received an infusion of autologous anti-CD19 CAR T cells (KYV-101), resulting in a reduction of GABAergic medication, as well as improvements in leg stiffness, gait, walking speed, and daily walking distance (138).

### 5.12 ANCA-associated vasculitis

In a 52-year-old male patient with severe, therapy-refractory proteinase-3 (PR3)- AAV, anti-CD19 CAR T-cell therapy induced both clinical remission and seroconversion of PR3-ANCA (139). Initial bone marrow biopsies, taken post-rituximab and before anti-CD19 CAR T-cell therapy, showed CD19^+^/CD20^-^ B cells, including plasma cells in bone marrow. However, a second biopsy after CAR T-cell therapy revealed CAR T cells and the absence of CD19+ B cells or CD19^low^ plasma cells in bone marrow, while CD138^+^/CD19^-^ plasma cells persisted. These findings confirm limited ability of rituximab to eliminate tissue-resident B cells in primary and secondary lymphoid organs and highlight effective B-cell depletion in the bone marrow following anti-CD19 CAR T-cell therapy.

### 5.13 Ulcerative colitis

There are two cases of IBD-like colitis following CAR T cell therapy for lymphoma have been reported, highlighting the need for close monitoring of lower gastrointestinal symptoms in patients undergoing CAR T-cell therapy (140, 141). More importantly, additional data is required to validate the effectiveness and safety of CAR-T therapy in patients with IBD.

## 6 Safety of CAR-T therapy

### 6.1 Risk of infection

As cytopenia and hypogammaglobulinemia are potential side effects of CAR T-cell therapy, infection has become an increasingly recognized complication, arising from both host-related factors and CAR T-associated factors (142). Like hematological malignancies, patients with IMIDs undergoing CAR T-cell therapy typically have relapsed/refractory disease with a history of multiple treatments, can lead to immune exhaustion, decreased bone marrow reserve with preexisting cytopenia, and delays in post-treatment immune recovery (143). Compared to patients undergoing stem cell therapy, post-CAR T-cell immune reconstitution is less well understood. Research on infection and immune recovery after CAR T-cell therapy is essential for developing effective prophylactic and treatment strategies for these patients (144). Hypogammaglobulinemia occurs in most patients after CAR T-cell therapy, requiring enhanced immunologic monitoring to identify those at high risk for severe infections and mortality who may benefit from immunoglobulin replacement (145).

### 6.2 Cytokine release syndrome and neurotoxicity syndrome

Two toxic effects of CAR T-cell therapy are of particular concern: cytokine release syndrome, which is mediated by CAR T-cell activation upon target cell engagement and the release of proinflammatory mediators such as IL-6, and neurotoxicity syndrome, which involves endothelial activation and disruption of the blood–brain barrier (146). Cytokine release syndrome rates in cancer range from 42% to 93% across all therapeutic cell products (146), however, in IMIDs, the occurrence was minimal, with no high-grade or prolonged bone marrow toxicity observed (10). More importantly, the key risk factor for both toxic effects in tumors is a high target cell burden, which is not present in IMIDs.

### 6.3 Secondary malignancy after CAR-T therapy

Although FDA adds boxed warning of secondary cancer to CAR T-cell therapies in hematologic malignancy, there is no solid evidence to support a higher risk of secondary cancer among IMID patients treated with CAR T than those treated with other therapies. A retrospective comparative cohort study showed a comparable safety profile of CAR T-cell therapy for cancer patients with and without pre-existing autoimmune or inflammatory disease (147). Notably, the DESCAR-T registry indicates a very low risk of T cell malignancy in patients with hematologic malignancies following CAR T-cell therapy, and integration site analysis suggests that the therapy may have contributed to the development of secondary T cell malignancy (148). Alternative factors, including prior multiple lines of treatment, immune dysfunction and clonal hematopoiesis of underlying disease, likely play a greater role than insertional mutagenesis in predisposing CAR-T-treated patients to secondary hematologic malignancies (148). Although the benefits of CAR T-cell therapy for IMID outweigh the risks, vigilant monitoring for secondary cancer remains essential.

## 7 Clinical trials of CAR-T for immune mediated inflammatory diseases

Currently, 23 trials are registered in the clinicaltrials.gov database to evaluate the efficacy of CAR-T therapy in IMIDs, including SLE, Sjögren’s syndrome, scleroderma, pemphigus vulgaris, neuromyelitis optica, and myasthenia gravis, among others (**Supplementary Table S1**). Emerging clinical results from recent EULAR, ACR, EHA meeting provide preliminary human data on how targeted cell therapy programs the immune system to durably reverse autoimmunity—an outcome previously unattainable with targeted biologic agents and stem cell therapies (**Supplementary Table S2**).

Several prospective clinical trials are underway to assess the safety and efficacy of CAR19-T cell therapy for SLE. An open-label, single-arm, multicenter phase 1/2 study (NCT05798117) provided preliminary data from the first three sentinel patients with severe refractory SLE who received YTB323, indicating favorable safety, CAR-T cell expansion, B cell depletion, and initial efficacy (103). Another pilot trial indicated that BCMA-CD19 CAR-T therapy in 13 patients with SLE or LN could eliminate autoantibodies, reset the B cell and humoral immune systems, and provide long-term, medication-free remission with a single dose (149).

## 8 Future directions and challenges

Building on advances in CAR T immunoengineering and insights gained from the use of conventional CAR T cell therapies in cancer, greater efforts have been directed toward targeting multiple factors implicated in the pathogenesis of IMIDs. Although the successful application of TyU19 and KYV-201 in IMIDs encourages the further exploration of allogeneic CAR-T therapy in patients with severe and refractory IMIDs (120), the optimal timing for allogeneic CAR-T therapy in autoimmunity requires further investigation due to the potential unknown short- and long-term safety risks and toxicity associated with off-the-shelf CAR-T therapies (150–152).

All commercially available CAR-T cell therapies express second-generation CARs containing a single costimulatory domain, whereas third-generation CAR-Ts, which incorporate two costimulatory domains, have demonstrated promising clinical efficacy and remarkably low procedure-specific toxicity in acute lymphoblastic leukemia (153). The efficacy of third-generation CAR-Ts in IMIDs requires further investigation. Many patients with B cell malignancies unfortunately fail to achieve durable responses to CAR-T therapy. Further research is needed to address resistance to CAR-T therapies, even though such resistance has not been observed in patients with IMIDs to date (154). The high costs and regulatory challenges of viral vectors in CAR T therapies have led to growing interest in non-viral gene delivery as a potential alternative (155). However, low knock-in efficiency remains a challenge in non-viral CAR-T cell production, underscoring the need for advancements in non-viral vector design and clinical manufacturing (156). As different IMIDs with shared cellular biology are often treated with similar therapies, more strategically designed cross-disease and cross-discipline basket trials should be implemented to mitigate the risk of failure in early-phase CAR-T cell therapy studies in IMIDs (157).

In comparison to targeted biologics and stem cell therapies, CAR-T cell therapy offers highly individualized characteristics, allowing for the precise recognition and targeting of immune-dysregulated cells, which enhances efficacy and reduces side effects. Although the outlook for CAR-T cell therapy in the treatment of IMIDs is highly optimistic, targeted cell therapies still face numerous challenges that limit their widespread translation and commercialization, including the optimization of proliferation and persistence, enhancement of potency, and development of effective, scalable manufacturing processes (158–161). Cutting-edge basic research, driven by immunoengineering approaches, could help address these challenges and pave the way for a new class of therapeutics focused on immunomodulation rather than immunosuppression (162, 163).

## 9 Conclusions

Despite advancements in targeted biologic therapies for IMIDs, challenges such as issues with long-term maintenance, non-responsiveness, and adverse side effects continue to pose significant barriers. Stem cell therapy is increasingly recognized as a standard salvage treatment, while CAR-T therapy holds promising potential for achieving long-lasting remissions. A clear understanding of the differences between these therapies is crucial for accurately evaluating the current status and future prospects of CAR-T therapy. Ongoing research is critical to addressing existing limitations and improving treatment outcomes for IMIDs.

## References

[1] Schett G McInnes IB Neurath MF. Reframing immune-mediated inflammatory diseases through signature cytokine hubs. N Engl J Med. (2021) 385(7):628–39. doi: 10.1056/NEJMra1909094 34379924

[2] Collaborators GI. Global, regional, and national incidence of six major immune-mediated inflammatory diseases: findings from the global burden of disease study 2019. EClinicalMedicine. (2023) 64:102193. doi: 10.1016/j.eclinm.2023.102193 37731935 PMC10507198

[3] Conrad N Misra S Verbakel JY Verbeke G Molenberghs G Taylor PN. Incidence, prevalence, and co-occurrence of autoimmune disorders over time and by age, sex, and socioeconomic status: a population-based cohort study of 22 million individuals in the UK. Lancet. (2023) 401(10391):1878–90. doi: 10.1016/S0140-6736(23)00457-9 37156255

[4] Gupta A Weinand K Nathan A Sakaue S Zhang MJ Accelerating Medicines Partnership RASLEP. Dynamic regulatory elements in single-cell multimodal data implicate key immune cell states enriched for autoimmune disease heritability. Nat Genet. (2023) 55(12):2200–10. doi: 10.1038/s41588-023-01577-7 PMC10787644 38036783

[5] McInnes IB Gravallese EM. Immune-mediated inflammatory disease therapeutics: past, present and future. Nat Rev Immunol. (2021) 21(10):680–6. doi: 10.1038/s41577-021-00603-1 PMC8436867 34518662

[6] Alexander T Greco R Snowden JA. Hematopoietic stem cell transplantation for autoimmune disease. Annu Rev Med. (2021) 72:215–28. doi: 10.1146/annurev-med-070119-115617 33106103

[7] Bertolino GM Maumus M Jorgensen C Noel D. Therapeutic potential in rheumatic diseases of extracellular vesicles derived from mesenchymal stromal cells. Nat Rev Rheumatol. (2023) 19(11):682–94. doi: 10.1038/s41584-023-01010-7 37666995

[8] Zaripova LN Midgley A Christmas SE Beresford MW Pain C Baildam EM. Mesenchymal stem cells in the pathogenesis and therapy of autoimmune and autoinflammatory diseases. Int J Mol Sci. (2023) 24(22). doi: 10.3390/ijms242216040 PMC10671211 38003230

[9] Mackensen A Müller F Mougiakakos D Böltz S Wilhelm A Aigner M. Anti-CD19 CAR t cell therapy for refractory systemic lupus erythematosus. Nat Med. (2022) 28(10):2124–32. doi: 10.1038/s41591-022-02017-5 36109639

[10] Müller F Taubmann J Bucci L Wilhelm A Bergmann C Völkl S. CD19 CAR t-cell therapy in autoimmune disease - a case series with follow-up. N Engl J Med. (2024) 390(8):687–700. doi: 10.1056/NEJMoa2308917 38381673

[11] Westin J Sehn LH. CAR t cells as a second-line therapy for large b-cell lymphoma: a paradigm shift? Blood. (2022) 139(18):2737–46. doi: 10.1182/blood.2022015789 35240677

[12] Kamdar M Solomon SR Arnason J Johnston PB Glass B Bachanova V. Lisocabtagene maraleucel versus standard of care with salvage chemotherapy followed by autologous stem cell transplantation as second-line treatment in patients with relapsed or refractory large b-cell lymphoma (TRANSFORM): results from an interim analysis of an open-label, randomised, phase 3 trial. Lancet. (2022) 399(10343):2294–308. doi: 10.1016/S0140-6736(22)00662-6 35717989

[13] Westin JR Oluwole OO Kersten MJ Miklos DB Perales MA Ghobadi A. Survival with axicabtagene ciloleucel in large b-cell lymphoma. N Engl J Med. (2023) 389(2):148–57. doi: 10.1056/NEJMoa2301665 37272527

[14] Solitano V Yuan Y Singh S Ma C Nardone OM Fiorino G. Efficacy and safety of advanced combination treatment in immune-mediated inflammatory disease: A systematic review and meta-analysis of randomized controlled trials. J Autoimmun. (2024) 149:103331. doi: 10.1016/j.jaut.2024.103331 39509741 PMC12186700

[15] Feldmann M Maini RN Soriano ER Strand V Takeuchi T. 25 years of biologic DMARDs in rheumatology. Nat Rev Rheumatol. (2023) 19(12):761–6. doi: 10.1038/s41584-023-01036-x 37919339

[16] Konzett V Smolen JS Nash P Aletaha D Winthrop K Dörner T. Efficacy of janus kinase inhibitors in immune-mediated inflammatory diseases-a systematic literature review informing the 2024 update of an international expert consensus statement. Ann Rheum Dis. (2025) 76(10):1560–5. doi: 10.1016/j.ard.2025.01.023 39934019

[17] Wijbrandts CA Tak PP. Prediction of response to targeted treatment in rheumatoid arthritis. Mayo Clin Proc. (2017) 92(7):1129–43. doi: 10.1016/j.mayocp.2017.05.009 28688467

[18] Tak PP. A personalized medicine approach to biologic treatment of rheumatoid arthritis: a preliminary treatment algorithm. Rheumatol (Oxford). (2012) 51(4):600–9. doi: 10.1093/rheumatology/ker300 PMC3306168 21890615

[19] Jamnitski A Bartelds GM Nurmohamed MT van Schouwenburg PA van Schaardenburg D Stapel SO. The presence or absence of antibodies to infliximab or adalimumab determines the outcome of switching to etanercept. Ann Rheum Dis. (2011) 70(2):284–8. doi: 10.1136/ard.2010.135111 21068090

[20] Paramarta JE Baeten DL. Adalimumab serum levels and antidrug antibodies towards adalimumab in peripheral spondyloarthritis: no association with clinical response to treatment or with disease relapse upon treatment discontinuation. Arthritis Res Ther. (2014) 16(4):R160. doi: 10.1186/ar4675 25074046 PMC4261980

[21] Jiang Y Chen Y Yu Q Shi Y. Biologic and small-molecule therapies for moderate-to-Severe psoriasis: Focus on psoriasis comorbidities. BioDrugs. (2023) 37(1):35–55. doi: 10.1007/s40259-022-00569-z 36592323 PMC9837020

[22] Quartuccio L Zabotti A Del Zotto S Zanier L De Vita S Valent F. Risk of serious infection among patients receiving biologics for chronic inflammatory diseases: Usefulness of administrative data. J Adv Res. (2019) 15:87–93. doi: 10.1016/j.jare.2018.09.003 30581616 PMC6300460

[23] Konzett V Smolen JS Nash P Winthrop K Aletaha D Dörner T. Safety of janus kinase inhibitors in immune-mediated inflammatory diseases-a systematic literature review informing the 2024 update of an international expert consensus statement. Ann Rheum Dis. (2025) S0003-4967(25):00080–9. doi: 10.1016/j.ard.2025.01.024 39934016

[24] Nash P Kerschbaumer A Dörner T Dougados M Fleischmann RM Geissler K. Points to consider for the treatment of immune-mediated inflammatory diseases with janus kinase inhibitors: a consensus statement. Ann Rheum Dis. (2021) 80(1):71–87. doi: 10.1136/annrheumdis-2020-218398 33158881 PMC7788060

[25] Schett G Nagy G Krönke G Mielenz D. B-cell depletion in autoimmune diseases. Ann Rheum Dis. (2024) 83(11):1409–20. doi: 10.1136/ard-2024-225727 38777374

[26] Stockfelt M Teng YKO Vital EM. Opportunities and limitations of b cell depletion approaches in SLE. Nat Rev Rheumatol. (2025) 21(2):111–26. doi: 10.1038/s41584-024-01210-9 39815102

[27] Humbel M Bellanger F Fluder N Horisberger A Suffiotti M Fenwick C. Restoration of NK cell cytotoxic function with elotuzumab and daratumumab promotes elimination of circulating plasma cells in patients with SLE. Front Immunol. (2021) 12:645478. doi: 10.3389/fimmu.2021.645478 33828555 PMC8019934

[28] Merrill JT Guthridge J Smith M June J Koumpouras F Machua W. Obexelimab in systemic lupus erythematosus with exploration of response based on gene pathway co-expression patterns: A double-blind, randomized, placebo-controlled, phase 2 trial. Arthritis Rheumatol. (2023) 75(12):2185–94. doi: 10.1002/art.42652 37459248

[29] Clowse ME Wallace DJ Furie RA Petri MA Pike MC Leszczyński P. Efficacy and safety of epratuzumab in moderately to severely active systemic lupus erythematosus: Results from two phase III randomized, double-blind, placebo-controlled trials. Arthritis Rheumatol. (2017) 69(2):362–75. doi: 10.1002/art.39856 PMC5299488 27598855

[30] Casulo C Maragulia J Zelenetz AD. Incidence of hypogammaglobulinemia in patients receiving rituximab and the use of intravenous immunoglobulin for recurrent infections. Clin Lymphoma Myeloma Leuk. (2013) 13(2):106–11. doi: 10.1016/j.clml.2012.11.011 PMC4035033 23276889

[31] Huang H Benoist C Mathis D. Rituximab specifically depletes short-lived autoreactive plasma cells in a mouse model of inflammatory arthritis. Proc Natl Acad Sci U S A. (2010) 107(10):4658–63. doi: 10.1073/pnas.1001074107 PMC2842072 20176942

[32] Chang HD Tokoyoda K Hoyer B Alexander T Khodadadi L Mei H. Pathogenic memory plasma cells in autoimmunity. Curr Opin Immunol. (2019) 61:86–91. doi: 10.1016/j.coi.2019.09.005 31675681 PMC6908965

[33] Lee S Ko Y Kim TJ. Homeostasis and regulation of autoreactive b cells. Cell Mol Immunol. (2020) 17(6):561–9. doi: 10.1038/s41423-020-0445-4 PMC7264189 32382130

[34] Winthrop KL Mease P Kerschbaumer A Voll RE Breedveld FC Smolen JS. Unmet need in rheumatology: reports from the advances in targeted therapies meeting, 2023. Ann Rheum Dis. (2024) 83(4):409–16. doi: 10.1136/ard-2023-224916 38123338

[35] Zeng L Liu C Wu Y Liu S Zheng Y Hao W. Efficacy and safety of mesenchymal stromal cell transplantation in the treatment of autoimmune and rheumatic immune diseases: a systematic review and meta-analysis of randomized controlled trials. Stem Cell Res Ther. (2025) 16(1):65. doi: 10.1186/s13287-025-04184-x 39934871 PMC11817852

[36] Zakrzewski W Dobrzyński M Szymonowicz M Rybak Z. Stem cells: past, present, and future. Stem Cell Res Ther. (2019) 10(1):68. doi: 10.1186/s13287-019-1165-5 30808416 PMC6390367

[37] Gilkeson GS. Safety and efficacy of mesenchymal stromal cells and other cellular therapeutics in rheumatic diseases in 2022: A review of what we know so far. Arthritis Rheumatol. (2022) 74(5):752–65. doi: 10.1002/art.42081 35128813

[38] Zeher M Papp G Nakken B Szodoray P. Hematopoietic stem cell transplantation in autoimmune disorders: From immune-regulatory processes to clinical implications. Autoimmun Rev. (2017) 16(8):817–25. doi: 10.1016/j.autrev.2017.05.020 28572052

[39] Alexander T Farge D Badoglio M Lindsay JO Muraro PA Snowden JA. Hematopoietic stem cell therapy for autoimmune diseases - clinical experience and mechanisms. J Autoimmun. (2018) 92:35–46. doi: 10.1016/j.jaut.2018.06.002 29934135

[40] Wang S Zhu R Li H Li J Han Q Zhao RC. Mesenchymal stem cells and immune disorders: from basic science to clinical transition. Front Med. (2019) 13(2):138–51. doi: 10.1007/s11684-018-0627-y 30062557

[41] McBride DA Jones RM Bottini N Shah NJ. The therapeutic potential of immunoengineering for systemic autoimmunity. Nat Rev Rheumatol. (2024) 20(4):203–15. doi: 10.1038/s41584-024-01084-x 38383732

[42] Konig MF. The rise of precision cellular therapies. Nat Rev Rheumatol. (2024) 20(2):69–70. doi: 10.1038/s41584-023-01073-6 38191701

[43] Abeles I Palma C Meednu N Payne AS Looney RJ Anolik JH. B cell-directed therapy in autoimmunity. Annu Rev Immunol. (2024) 42(1):103–26. doi: 10.1146/annurev-immunol-083122-044829 38011889

[44] Zhou J Lei B Shi F Luo X Wu K Xu Y. CAR t-cell therapy for systemic lupus erythematosus: current status and future perspectives. Front Immunol. (2024) 15:1476859. doi: 10.3389/fimmu.2024.1476859 39749335 PMC11694027

[45] Sosnoski HM Posey AD Jr.. Therapeutic intersections: Expanding benefits of CD19 CAR t cells from cancer to autoimmunity. Cell Stem Cell. (2024) 31(4):437–8. doi: 10.1016/j.stem.2024.03.006 38579681

[46] Chung JB Brudno JN Borie D Kochenderfer JN. Chimeric antigen receptor t cell therapy for autoimmune disease. Nat Rev Immunol. (2024) 24(11):830–45. doi: 10.1038/s41577-024-01035-3 PMC12176013 38831163

[47] Baker DJ June CH. Off-the-shelf CAR-t cells could prove paradigm shifting for autoimmune diseases. Cell. (2024) 187(18):4826–8. doi: 10.1016/j.cell.2024.07.056 39241743

[48] Cappell KM Kochenderfer JN. A comparison of chimeric antigen receptors containing CD28 versus 4-1BB costimulatory domains. Nat Rev Clin Oncol. (2021) 18(11):715–27. doi: 10.1038/s41571-021-00530-z 34230645

[49] Schett G Mackensen A Mougiakakos D. CAR t-cell therapy in autoimmune diseases. Lancet. (2023) 402(10416):2034–44. doi: 10.1016/s0140-6736(23)01126-1 37748491

[50] Larson RC Maus MV. Recent advances and discoveries in the mechanisms and functions of CAR t cells. Nat Rev Cancer. (2021) 21(3):145–61. doi: 10.1038/s41568-020-00323-z PMC8353572 33483715

[51] Liu Z Xiao Y Lyu J Jing D Liu L Fu Y. The expanded application of CAR-t cell therapy for the treatment of multiple non-tumoral diseases. Protein Cell. (2024) 15(9):633–41. doi: 10.1093/procel/pwad061 PMC11365555 38146589

[52] Dhodapkar KM Cohen AD Kaushal A Garfall AL Manalo RJ Carr AR. Changes in bone marrow tumor and immune cells correlate with durability of remissions following BCMA CAR t therapy in myeloma. Blood Cancer Discovery. (2022) 3(6):490–501. doi: 10.1158/2643-3230.Bcd-22-0018 36026513 PMC9627239

[53] Biondi M Tettamanti S Galimberti S Cerina B Tomasoni C Piazza R. Selective homing of CAR-CIK cells to the bone marrow niche enhances control of the acute myeloid leukemia burden. Blood. (2023) 141(21):2587–98. doi: 10.1182/blood.2022018330 PMC10646802 36787509

[54] Dingfelder J Aigner M Taubmann J Minopoulou I Park S Kaplan CD. Fully human anti-CD19 CAR t cells derived from systemic lupus erythematosus patients exhibit cytotoxicity with reduced inflammatory cytokine production. Transplant Cell Ther. (2024) 30(6):582. doi: 10.1016/j.jtct.2024.03.023 38548226

[55] Melenhorst JJ Chen GM Wang M Porter DL Chen C Collins MA. Decade-long leukaemia remissions with persistence of CD4(+) CAR t cells. Nature. (2022) 602(7897):503–9. doi: 10.1038/s41586-021-04390-6 PMC9166916 35110735

[56] Odak I Bayir LM Riemann L Sikora R Schneider J Xiao Y. Brief research report: in-depth immunophenotyping reveals stability of CD19 CAR t-cells over time. Front Immunol. (2024) 15:1298598. doi: 10.3389/fimmu.2024.1298598 38318174 PMC10839090

[57] Qin C Dong MH Zhou LQ Wang W Cai SB You YF. Single-cell analysis of refractory anti-SRP necrotizing myopathy treated with anti-BCMA CAR-t cell therapy. Proc Natl Acad Sci U S A. (2024) 121(6):e2315990121. doi: 10.1073/pnas.2315990121 38289960 PMC10861907

[58] Tian DS Qin C Dong MH Heming M Zhou LQ Wang W. B cell lineage reconstitution underlies CAR-t cell therapeutic efficacy in patients with refractory myasthenia gravis. EMBO Mol Med. (2024) 16(4):966–87. doi: 10.1038/s44321-024-00043-z PMC11018773 38409527

[59] Tang L Huang ZP Mei H Hu Y. Insights gained from single-cell analysis of chimeric antigen receptor t-cell immunotherapy in cancer. Mil Med Res. (2023) 10(1):52. doi: 10.1186/s40779-023-00486-4 37941075 PMC10631149

[60] Li X Guo X Zhu Y Wei G Zhang Y Li X. Single-cell transcriptomic analysis reveals BCMA CAR-t cell dynamics in a patient with refractory primary plasma cell leukemia. Mol Ther. (2021) 29(2):645–57. doi: 10.1016/j.ymthe.2020.11.028 PMC7854300 33278564

[61] Qin C Zhang M Mou DP Zhou LQ Dong MH Huang L. Single-cell analysis of anti-BCMA CAR t cell therapy in patients with central nervous system autoimmunity. Sci Immunol. (2024) 9(95):eadj9730. doi: 10.1126/sciimmunol.adj9730 38728414

[62] Claus M Freitag M Ewald M Rodon L Deicher F Watzl C. Immunological effects of CD19. CAR-t cell therapy in systemic sclerosis: an extended case study. Arthritis Res Ther. (2024) 26(1):211. doi: 10.1186/s13075-024-03451-1 39673062 PMC11639114

[63] Kansal R Richardson N Neeli I Khawaja S Chamberlain D Ghani M. Sustained b cell depletion by CD19-targeted CAR t cells is a highly effective treatment for murine lupus. Sci Transl Med. (2019) 11(482):eaav1648. doi: 10.1126/scitranslmed.aav1648 30842314 PMC8201923

[64] Jin X Xu Q Pu C Zhu K Lu C Jiang Y. Therapeutic efficacy of anti-CD19 CAR-t cells in a mouse model of systemic lupus erythematosus. Cell Mol Immunol. (2021) 18(8):1896–903. doi: 10.1038/s41423-020-0472-1 PMC8322088 32472023

[65] Uppin V Gibbons H Troje M Feinberg D Webber BR Moriarity BS. CAR-t cell targeting three receptors on autoreactive b cells for systemic lupus erythematosus therapy. J Autoimmun. (2025) 151:103369. doi: 10.1016/j.jaut.2025.103369 39832454

[66] Lodka D Zschummel M Bunse M Rousselle A Sonnemann J Kettritz R. CD19-targeting CAR t cells protect from ANCA-induced acute kidney injury. Ann Rheum Dis. (2024) 83(4):499–507. doi: 10.1136/ard-2023-224875 38182404 PMC10958264

[67] Fransson M Piras E Burman J Nilsson B Essand M Lu B. CAR/FoxP3-engineered t regulatory cells target the CNS and suppress EAE upon intranasal delivery. J Neuroinflammation. (2012) 9:112. doi: 10.1186/1742-2094-9-112 22647574 PMC3403996

[68] Mitsdoerffer M Di Liberto G Dötsch S Sie C Wagner I Pfaller M. Formation and immunomodulatory function of meningeal b cell aggregates in progressive CNS autoimmunity. Brain. (2021) 144(6):1697–710. doi: 10.1093/brain/awab093 33693558

[69] Gupta S Simic M Sagan SA Shepherd C Duecker J Sobel RA. CAR-t cell-mediated b-cell depletion in central nervous system autoimmunity. Neurol Neuroimmunol Neuroinflamm. (2023) 10(2):e200080. doi: 10.1212/nxi.0000000000200080 36657993 PMC9853314

[70] Siegler EL Wang P. Preclinical models in chimeric antigen receptor-engineered t-cell therapy. Hum Gene Ther. (2018) 29(5):534–46. doi: 10.1089/hum.2017.243 29390873

[71] Reincke SM von Wardenburg N Homeyer MA Kornau HC Spagni G Li LY. Chimeric autoantibody receptor t cells deplete NMDA receptor-specific b cells. Cell. (2023) 186(23):5084–97.e18. doi: 10.1016/j.cell.2023.10.001 37918394

[72] Moorman CD Yu S Briseno CG Phee H Sahoo A Ramrakhiani A. CAR-t cells and CAR-tregs targeting conventional type-1 dendritic cell suppress experimental autoimmune encephalomyelitis. Front Immunol. (2023) 14:1235222. doi: 10.3389/fimmu.2023.1235222 37965348 PMC10641730

[73] Zhu HX Yang SH Gao CY Bian ZH Chen XM Huang RR. Targeting pathogenic CD8(+) tissue-resident t cells with chimeric antigen receptor therapy in murine autoimmune cholangitis. Nat Commun. (2024) 15(1):2936. doi: 10.1038/s41467-024-46654-5 38580644 PMC10997620

[74] Duan H Jiang Z Chen L Bai X Cai H Yang X. TSHR-based chimeric antigen receptor t cell specifically deplete auto-reactive b lymphocytes for treatment of autoimmune thyroid disease. Int Immunopharmacol. (2023) 124(Pt A):110873. doi: 10.1016/j.intimp.2023.110873 37690235

[75] Fishman S Lewis MD Siew LK De Leenheer E Kakabadse D Davies J. Adoptive transfer of mRNA-transfected t cells redirected against diabetogenic CD8 t cells can prevent diabetes. Mol Ther. (2017) 25(2):456–64. doi: 10.1016/j.ymthe.2016.12.007 PMC5368593 28109957

[76] Kobayashi S Thelin MA Parrish HL Deshpande NR Lee MS Karimzadeh A. A biomimetic five-module chimeric antigen receptor ((5M)CAR) designed to target and eliminate antigen-specific t cells. Proc Natl Acad Sci U S A. (2020) 117(46):28950–9. doi: 10.1073/pnas.2012495117 PMC7682351 33139567

[77] Radichev IA Yoon J Scott DW Griffin K Savinov AY. Towards antigen-specific tregs for type 1 diabetes: Construction and functional assessment of pancreatic endocrine marker, HPi2-based chimeric antigen receptor. Cell Immunol. (2020) 358:104224. doi: 10.1016/j.cellimm.2020.104224 33068914 PMC7655659

[78] Zhang L Sosinowski T Cox AR Cepeda JR Sekhar NS Hartig SM. Chimeric antigen receptor (CAR) t cells targeting a pathogenic MHC class II:peptide complex modulate the progression of autoimmune diabetes. J Autoimmun. (2019) 96:50–8. doi: 10.1016/j.jaut.2018.08.004 PMC6541442 30122420

[79] Tenspolde M Zimmermann K Weber LC Hapke M Lieber M Dywicki J. Regulatory t cells engineered with a novel insulin-specific chimeric antigen receptor as a candidate immunotherapy for type 1 diabetes. J Autoimmun. (2019) 103:102289. doi: 10.1016/j.jaut.2019.05.017 31176558

[80] Spanier JA Fung V Wardell CM Alkhatib MH Chen Y Swanson LA. Tregs with an MHC class II peptide-specific chimeric antigen receptor prevent autoimmune diabetes in mice. J Clin Invest. (2023) 133(18). doi: 10.1172/jci168601 PMC10503798 37561596

[81] Parvathaneni K Scott DW. Engineered FVIII-expressing cytotoxic t cells target and kill FVIII-specific b cells *in vitro* and in vivo. Blood Adv. (2018) 2(18):2332–40. doi: 10.1182/bloodadvances.2018018556 PMC6156881 30232086

[82] Skuljec J Chmielewski M Happle C Habener A Busse M Abken H. Chimeric antigen receptor-redirected regulatory t cells suppress experimental allergic airway inflammation, a model of asthma. Front Immunol. (2017) 8:1125. doi: 10.3389/fimmu.2017.01125 28955341 PMC5600908

[83] Ward DE Fay BL Adejuwon A Han H Ma Z. Chimeric antigen receptors based on low affinity mutants of FcepsilonRI re-direct t cell specificity to cells expressing membrane IgE. Front Immunol. (2018) 9:2231. doi: 10.3389/fimmu.2018.02231 30364107 PMC6191488

[84] Jin G Liu Y Wang L He Z Zhao X Ma Y. A single infusion of engineered long-lived and multifunctional t cells confers durable remission of asthma in mice. Nat Immunol. (2024) 25(6):1059–72. doi: 10.1038/s41590-024-01834-9 38802511

[85] Aghajanian H Kimura T Rurik JG Hancock AS Leibowitz MS Li L. Targeting cardiac fibrosis with engineered t cells. Nature. (2019) 573(7774):430–3. doi: 10.1038/s41586-019-1546-z PMC6752964 31511695

[86] Rurik JG Tombácz I Yadegari A Méndez Fernández PO Shewale SV Li L. CAR t cells produced *in vivo* to treat cardiac injury. Science. (2022) 375(6576):91–6. doi: 10.1126/science.abm0594 PMC9983611 34990237

[87] Oh S Mao X Manfredo-Vieira S Lee J Patel D Choi EJ. Precision targeting of autoantigen-specific b cells in muscle-specific tyrosine kinase myasthenia gravis with chimeric autoantibody receptor t cells. Nat Biotechnol. (2023) 41(9):1229–38. doi: 10.1038/s41587-022-01637-z PMC10354218 36658341

[88] Proby CM Ota T Suzuki H Koyasu S Gamou S Shimizu N. Development of chimeric molecules for recognition and targeting of antigen-specific b cells in pemphigus vulgaris. Br J Dermatol. (2000) 142(2):321–30. doi: 10.1046/j.1365-2133.2000.03328.x 10730768

[89] Ellebrecht CT Bhoj VG Nace A Choi EJ Mao X Cho MJ. Reengineering chimeric antigen receptor t cells for targeted therapy of autoimmune disease. Science. (2016) 353(6295):179–84. doi: 10.1126/science.aaf6756 PMC5343513 27365313

[90] Avouac J Cauvet A Orvain C Boulch M Tilotta F Tu L. Effects of b cell depletion by CD19-targeted chimeric antigen receptor t cells in a murine model of systemic sclerosis. Arthritis Rheumatol. (2024) 76(2):268–78. doi: 10.1002/art.42677 37610259

[91] Thurlings RM Vos K Wijbrandts CA Zwinderman AH Gerlag DM Tak PP. Synovial tissue response to rituximab: mechanism of action and identification of biomarkers of response. Ann Rheum Dis. (2008) 67(7):917–25. doi: 10.1136/ard.2007.080960 PMC2564787 17965121

[92] Eferl R Hasselblatt P Rath M Popper H Zenz R Komnenovic V. Development of pulmonary fibrosis through a pathway involving the transcription factor fra-2/AP-1. Proc Natl Acad Sci U S A. (2008) 105(30):10525–30. doi: 10.1073/pnas.0801414105 PMC2492511 18641127

[93] Sun Y Huang S Zhang B Peng Y Lu H Jia Y. Efficacy and safety of anti-CD19 CAR-t in a mouse model of IgG4-related disease. Int Immunopharmacol. (2025) 145:113779. doi: 10.1016/j.intimp.2024.113779 39672025

[94] Blat D Zigmond E Alteber Z Waks T Eshhar Z. Suppression of murine colitis and its associated cancer by carcinoembryonic antigen-specific regulatory t cells. Mol Ther. (2014) 22(5):1018–28. doi: 10.1038/mt.2014.41 PMC4015241 24686242

[95] Bachanova V Nachman PH. Two for one? CAR-t therapy for lymphoma benefits concurrent autoimmune disorders. Bone Marrow Transplant. (2023) 58(11):1175–6. doi: 10.1038/s41409-023-02084-3 PMC10980181 37598289

[96] Schmelz JL Navsaria L Goswamy R Chuang HH Miranda RN Lee HJ. Chimeric antigen receptor t-cell therapy's role in antiphospholipid syndrome: a case report. Br J Haematol. (2020) 188(3):e5–8. doi: 10.1111/bjh.16266 31721157

[97] Zhang W Feng J Cinquina A Wang Q Xu H Zhang Q. Treatment of systemic lupus erythematosus using BCMA-CD19 compound CAR. Stem Cell Rev Rep. (2021) 17(6):2120–3. doi: 10.1007/s12015-021-10251-6 PMC8599262 34458965

[98] Wang J Alkrekshi A Dasari S Lin HC Elantably D Armashi ARA. CD19-targeted chimeric antigen receptor t-cell therapy in patients with concurrent b-cell non-hodgkin lymphoma and rheumatic autoimmune diseases: a propensity score matching study. Bone Marrow Transplant. (2023) 58(11):1223–8. doi: 10.1038/s41409-023-02086-1 37604871

[99] Mougiakakos D Krönke G Völkl S Kretschmann S Aigner M Kharboutli S. CD19-targeted CAR t cells in refractory systemic lupus erythematosus. N Engl J Med. (2021) 385(6):567–9. doi: 10.1056/NEJMc2107725 34347960

[100] Nunez D Patel D Volkov J Wong S Vorndran Z Müller F. Cytokine and reactivity profiles in SLE patients following anti-CD19 CART therapy. Mol Ther Methods Clin Dev. (2023) 31:101104. doi: 10.1016/j.omtm.2023.08.023 37744005 PMC10514439

[101] Boulougoura A Gendelman H Surmachevska N Kyttaris VC. Journal club: Anti-CD19 chimeric antigen receptor t cell therapy for refractory systemic lupus erythematosus. ACR Open Rheumatol. (2023) 5(11):624–8. doi: 10.1002/acr2.11614 PMC10642250 37766597

[102] Li M Zhang Y Jiang N Ning C Wang Q Xu D. Anti-CD19 CAR t cells in refractory immune thrombocytopenia of SLE. N Engl J Med. (2024) 391(4):376–8. doi: 10.1056/NEJMc2403743 39047248

[103] Cortés Hernández J BP Linares Alberich M Fischer O Kovacs B Calzascia T. An open-label, multicenter, phase 1/2 study to assess safety, efficacy and cellular kinetics of YTB323, a rapid manufacturing CAR-t cell therapy targeting CD19 on b cells, for severe refractory systemic lupus erythematosus: Preliminary results [abstract]. Arthritis Rheumatol. (2023) 75(suppl 9).

[104] Cortés-Hernández J Barba P Alvaro-Gracia JM Kwon M Weinmann-Menke J Wagner-Drouet E. POS0046.Preliminary results of an open-label, multicentre, phase 1/2 study to assess safety, efficacy and cellular kinetics of Ytb323 (Rapcabtagene autoleucel), a rapidly manufactured CAR t-cell therapy targeting CD19 on b cells, for severe refractory systemic lupus erythematosus. Ann Rheum Dis. (2024) 83:327–8. doi: 10.1136/annrheumdis-2024-eular.1768

[105] Krickau T Naumann-Bartsch N Aigner M Kharboutli S Kretschmann S Spoerl S. CAR t-cell therapy rescues adolescent with rapidly progressive lupus nephritis from haemodialysis. Lancet. (2024) 403(10437):1627–30. doi: 10.1016/s0140-6736(24)00424-0 38642568

[106] Emiliano M Claudia B Pietro M Patricia Moran A Rebecca N Mattia A. P101 anti-CD19 CAR-t cell therapy for refractory childhood-onset systemic lupus erythematosus. Lupus Sci Med. (2024) 11. doi: 10.1136/lupus-2024-el.155

[107] He X Hu B Zhang Y Liu F Li Q Zheng C. Treatment of two pediatric patients with refractory systemic lupus erythematosus using CD19-targeted CAR t-cells. Autoimmun Rev. (2025) 24(1):103692. doi: 10.1016/j.autrev.2024.103692 39561867

[108] Wang W He S Zhang W Zhang H DeStefano VM Wada M. BCMA-CD19 compound CAR t cells for systemic lupus erythematosus: a phase 1 open-label clinical trial. Ann Rheum Dis. (2024) 83(10):1304–14. doi: 10.1136/ard-2024-225785 38777376

[109] Hagen M Müller F Wirsching A Kharboutli S Spörl S Aigner M. Treatment of CNS systemic lupus erythematosus with CD19 CAR t cells. Lancet. (2024) 404(10468):2158–60. doi: 10.1016/s0140-6736(24)02265-7 39615991

[110] Ye Y Gaugler B Mohty M Malard F. Plasmacytoid dendritic cell biology and its role in immune-mediated diseases. Clin Transl Immunol. (2020) 9(5):e1139. doi: 10.1002/cti2.1139 PMC7248678 32489664

[111] Werth VP Furie RA Romero-Diaz J Navarra S Kalunian K van Vollenhoven RF. Trial of anti-BDCA2 antibody litifilimab for cutaneous lupus erythematosus. N Engl J Med. (2022) 387(4):321–31. doi: 10.1056/NEJMoa2118024 35939578

[112] Furie RA van Vollenhoven RF Kalunian K Navarra S Romero-Diaz J Werth VP. Trial of anti-BDCA2 antibody litifilimab for systemic lupus erythematosus. N Engl J Med. (2022) 387(10):894–904. doi: 10.1056/NEJMoa2118025 36069871

[113] Cael B Bole-Richard E Galaine J Adotevi O Garnache-Ottou F Aubin F. Production of functional plasmacytoid dendritic cells - targeted chimeric antigen receptor t cells from patients with immune-mediated inflammatory diseases. Br J Dermatol. (2023) 189(2):234–6. doi: 10.1093/bjd/ljad105 37022745

[114] Friedberg E Wohlfarth P Schiefer AI Skrabs C Pickl WF Worel N. Disappearance of antiphospholipid antibodies after anti-CD19 chimeric antigen receptor t-cell therapy of b-cell lymphoma in a patient with systemic lupus erythematosus and antiphospholipid syndrome. J Thromb Haemost. (2025) 23(1):262–6. doi: 10.1016/j.jtha.2024.09.024 39393780

[115] Müller F Boeltz S Knitza J Aigner M Völkl S Kharboutli S. CD19-targeted CAR t cells in refractory antisynthetase syndrome. Lancet. (2023) 401(10379):815–8. doi: 10.1016/s0140-6736(23)00023-5 36930673

[116] Pecher AC Hensen L Klein R Schairer R Lutz K Atar D. CD19-targeting CAR t cells for myositis and interstitial lung disease associated with antisynthetase syndrome. Jama. (2023) 329(24):2154–62. doi: 10.1001/jama.2023.8753 PMC10300719 37367976

[117] Taubmann J Knitza J Müller F Völkl S Aigner M Kleyer A. Rescue therapy of antisynthetase syndrome with CD19-targeted CAR-t cells after failure of several b-cell depleting antibodies. Rheumatol (Oxford). (2024) 63(1):e12–e4. doi: 10.1093/rheumatology/kead330 PMC10765150 37432378

[118] Nicolai R Merli P Moran Alvarez P Bracaglia C Del Bufalo F Marasco E. Autologous CD19-targeting CAR t cells in a patient with refractory juvenile dermatomyositis. Arthritis Rheumatol. (2024) 76(10):1560–5. doi: 10.1002/art.42933 38924652

[119] Volkov J Nunez D Mozaffar T Stadanlick J Werner M Vorndran Z. Case study of CD19 CAR t therapy in a subject with immune-mediate necrotizing myopathy treated in the RESET-myositis phase I/II trial. Mol Ther. (2024) 32(11):3821–8. doi: 10.1016/j.ymthe.2024.09.009 PMC11573600 39245937

[120] Wang X Wu X Tan B Zhu L Zhang Y Lin L. Allogeneic CD19-targeted CAR-t therapy in patients with severe myositis and systemic sclerosis. Cell. (2024) 187(18):4890–904.e9. doi: 10.1016/j.cell.2024.06.027 39013470

[121] Bergmann C Müller F Distler JHW Györfi AH Völkl S Aigner M. Treatment of a patient with severe systemic sclerosis (SSc) using CD19-targeted CAR t cells. Ann Rheum Dis. (2023) 82(8):1117–20. doi: 10.1136/ard-2023-223952 PMC10359520 37147112

[122] Merkt W Freitag M Claus M Kolb P Falcone V Röhrich M. Third-generation CD19.CAR-t cell-containing combination therapy in Scl70+ systemic sclerosis. Ann Rheum Dis. (2024) 83(4):543–6. doi: 10.1136/ard-2023-225174 PMC10958299 38135464

[123] Müller F Mackensen A Schett G. CAR t-cell therapy in autoimmune disease. Reply. N Engl J Med. (2024) 390(17):1631–2. doi: 10.1056/NEJMc2403705 38692300

[124] Auth J Müller F Völkl S Bayerl N Distler JHW Tur C. CD19-targeting CAR t-cell therapy in patients with diffuse systemic sclerosis: a case series. Lancet Rheumatol. (2025) 7(2):e83–93. doi: 10.1016/s2665-9913(24)00282-0 39542003

[125] Zhang B Wang Y Yuan Y Sun J Liu L Huang D. *In vitro* elimination of autoreactive b cells from rheumatoid arthritis patients by universal chimeric antigen receptor t cells. Ann Rheum Dis. (2021) 80(2):176–84. doi: 10.1136/annrheumdis-2020-217844 32998865

[126] Haghikia A Hegelmaier T Wolleschak D Böttcher M Pappa V Motte J. Clinical efficacy and autoantibody seroconversion with CD19-CAR t cell therapy in a patient with rheumatoid arthritis and coexisting myasthenia gravis. Ann Rheum Dis. (2024) 83(11):1597–8. doi: 10.1136/ard-2024-226017 38937071

[127] Albach FN Minopoulou I Wilhelm A Biesen R Kleyer A Wiebe E. Targeting autoimmunity with CD19-CAR t cell therapy: efficacy and seroconversion in diffuse systemic sclerosis and rheumatoid arthritis. Rheumatol (Oxford). doi: 10.1093/rheumatology/keaf077 39928370

[128] Masihuddin A Liebowitz J Reshef R Bathon J. Remission of lymphoma & rheumatoid arthritis following anti-CD19 chimeric antigen receptor t cell therapy for diffuse large b-cell lymphoma. Rheumatol (Oxford). (2024), keae714. doi: 10.1093/rheumatology/keae714 39718785

[129] Lidar M Rimar D David P Jacoby E Shapira-Frommer R Itzhaki O. CD-19 CAR-t cells for polyrefractory rheumatoid arthritis. Ann Rheum Dis. (2025) 84(2):370–2. doi: 10.1136/ard-2024-226437 39919909

[130] Li Y Li S Zhao X Sheng J Xue L Schett G. Fourth-generation chimeric antigen receptor t-cell therapy is tolerable and efficacious in treatment-resistant rheumatoid arthritis. Cell Res. (2025) 35(3):220–3. doi: 10.1038/s41422-024-01068-2 PMC11909189 39779933

[131] Lee J Lundgren DK Mao X Manfredo-Vieira S Nunez-Cruz S Williams EF. Antigen-specific b cell depletion for precision therapy of mucosal pemphigus vulgaris. J Clin Invest. (2020) 130(12):6317–24. doi: 10.1172/JCI138416 PMC7685721 32817591

[132] Fischbach F Richter J Pfeffer LK Fehse B Berger SC Reinhardt S. CD19-targeted chimeric antigen receptor t cell therapy in two patients with multiple sclerosis. Med. (2024) 5(6):550–8.e2. doi: 10.1016/j.medj.2024.03.002 38554710

[133] Mullard A. CAR-t therapy for multiple sclerosis enters US trials for first time. Nature. (2024). doi: 10.1038/d41586-024-00470-5 38396094

[134] Qin C Tian DS Zhou LQ Shang K Huang L Dong MH. Anti-BCMA CAR t-cell therapy CT103A in relapsed or refractory AQP4-IgG seropositive neuromyelitis optica spectrum disorders: phase 1 trial interim results. Signal Transduct Target Ther. (2023) 8(1):5. doi: 10.1038/s41392-022-01278-3 36596762 PMC9810610

[135] Granit V Benatar M Kurtoglu M Miljković MD Chahin N Sahagian G. Safety and clinical activity of autologous RNA chimeric antigen receptor t-cell therapy in myasthenia gravis (MG-001): a prospective, multicentre, open-label, non-randomised phase 1b/2a study. Lancet Neurol. (2023) 22(7):578–90. doi: 10.1016/s1474-4422(23)00194-1 PMC10416207 37353278

[136] Haghikia A Hegelmaier T Wolleschak D Böttcher M Desel C Borie D. Anti-CD19 CAR t cells for refractory myasthenia gravis. Lancet Neurol. (2023) 22(12):1104–5. doi: 10.1016/s1474-4422(23)00375-7 37977704

[137] Motte J Sgodzai M Schneider-Gold C Steckel N Mika T Hegelmaier T. Treatment of concomitant myasthenia gravis and lambert-eaton myasthenic syndrome with autologous CD19-targeted CAR t cells. Neuron. (2024) 112(11):1757–63.e2. doi: 10.1016/j.neuron.2024.04.014 38697115

[138] Faissner S Motte J Sgodzai M Geis C Haghikia A Mougiakakos D. Successful use of anti-CD19 CAR t cells in severe treatment-refractory stiff-person syndrome. Proc Natl Acad Sci U S A. (2024) 121(26):e2403227121. doi: 10.1073/pnas.2403227121 38885382 PMC11214089

[139] Minopoulou I Wilhelm A Albach F Kleyer A Wiebe E Schallenberg S. Anti-CD19 CAR t cell therapy induces antibody seroconversion and complete b cell depletion in the bone marrow of a therapy-refractory patient with ANCA-associated vasculitis. Ann Rheum Dis. (2025) 84(3):e4–7. doi: 10.1016/j.ard.2025.01.008 39893102

[140] Hashim A Patten PEM Kuhnl A Ooft ML Hayee B Sanderson R. Colitis after CAR t-cell therapy for refractory large b-cell lymphoma responds to anti-integrin therapy. Inflammation Bowel Dis. (2021) 27(4):e45–e6. doi: 10.1093/ibd/izaa320 33252123

[141] Zundler S Vitali F Kharboutli S Volkl S Polifka I Mackensen A. Case report: IBD-like colitis following CAR t cell therapy for diffuse large b cell lymphoma. Front Oncol. (2023) 13:1149450. doi: 10.3389/fonc.2023.1149450 37284193 PMC10240064

[142] Hill JA Seo SK. How i prevent infections in patients receiving CD19-targeted chimeric antigen receptor t cells for b-cell malignancies. Blood. (2020) 136(8):925–35. doi: 10.1182/blood.2019004000 PMC7441168 32582924

[143] Wudhikarn K Perales MA. Infectious complications, immune reconstitution, and infection prophylaxis after CD19 chimeric antigen receptor t-cell therapy. Bone Marrow Transplant. (2022) 57(10):1477–88. doi: 10.1038/s41409-022-01756-w PMC9285870 35840746

[144] García-Poutón N Peyrony O Chumbita M Aiello F Monzo P Gallardo-Pizarro A. Post-CART-T cell infection: Etiology, pathogenesis, and therapeutic approaches. Rev Esp Quimioter. (2023) 36(Suppl 1):52–3. doi: 10.37201/req/s01.12.2023 PMC10793555 37997872

[145] Sutherland NM Zhou B Zhang L Ong MS Hong JS Pak A. Association of CD19(+)-targeted chimeric antigen receptor (CAR) t-cell therapy with hypogammaglobulinemia, infection, and mortality. J Allergy Clin Immunol. (2025) 155(2):605–15. doi: 10.1016/j.jaci.2024.10.021 PMC11805655 39505278

[146] Chohan KL Siegler EL Kenderian SS. CAR-t cell therapy: the efficacy and toxicity balance. Curr Hematol Malig Rep. (2023) 18(2):9–18. doi: 10.1007/s11899-023-00687-7 36763238 PMC10505056

[147] Vanni KMM McCarter KR Wang X Duffy C Cruz JPD Wobma H. Safety of CAR t-cell therapy for cancer in pre-existing autoimmune or inflammatory disease: a retrospective comparative cohort study. Lancet Rheumatol. (2025) S2665-9913(24):00402–8. doi: 10.1016/s2665-9913(24)00402-8 39889722

[148] Dulery R Guiraud V Choquet S Thieblemont C Bachy E Barete S. T cell malignancies after CAR t cell therapy in the DESCAR-t registry. Nat Med. (2025). doi: 10.1038/s41591-024-03458-w 39779930

[149] Yuan Y HS Zhang W Zhang H DeStefano V Wada M. Novel BCMA-CD19 compound CAR-t (cCAR) targets b cells and plasma cells achieving immune reset and eliminates all autoantibodies in systemic lupus erythematosus (SLE) and lupus nephritis (LN) patients resulting in long-term, medication-free remission [abstract]. . Arthritis Rheumatol. (2023) 75(suppl 9).

[150] . Available at: https://kyvernatx.com/our-pipeline-expanded-access/ (Accessed October 30, 2024).

[151] Artiva biotherapeutics receives FDA fast track designation for AlloNK® in lupus nephritis. . Available at: https://www.artivabio.com/artiva-biotherapeutics-receives-fda-fast-track-designation-for-allonk-in-lupus-nephritis/ (Accessed October 30, 2024).

[152] Mahne A Kwong B Cheng J Wang J Banuelos J Starokadomskyy P. POS0462.Preclinical development of KYV-201, an investigational allogeneic anti-CD19 CAR-t cell for the treatment of autoimmune disease. Ann Rheum Dis. (2024) 83:1129–30. doi: 10.1136/annrheumdis-2024-eular.2343

[153] Schubert ML Schmitt A Huckelhoven-Krauss A Neuber B Kunz A Waldhoff P. Treatment of adult ALL patients with third-generation CD19-directed CAR t cells: results of a pivotal trial. J Hematol Oncol. (2023) 16(1):79. doi: 10.1186/s13045-023-01470-0 37481608 PMC10363324

[154] Singh N Orlando E Xu J Xu J Binder Z Collins MA. Mechanisms of resistance to CAR t cell therapies. Semin Cancer Biol. (2020) 65:91–8. doi: 10.1016/j.semcancer.2019.12.002 PMC7684646 31866478

[155] Balke-Want H Keerthi V Cadinanos-Garai A Fowler C Gkitsas N Brown AK. Non-viral chimeric antigen receptor (CAR) t cells going viral. Immunooncol Technol. (2023) 18:100375. doi: 10.1016/j.iotech.2023.100375 37124148 PMC10139995

[156] Enriquez-Rodriguez L Attia N Gallego I Mashal M Maldonado I Puras G. Expanding the horizon of transient CAR t therapeutics using virus-free technology. Biotechnol Adv. (2024) 72:108350. doi: 10.1016/j.biotechadv.2024.108350 38537878

[157] Hosack T Thomas T Ravindran R Uhlig HH Travis SPL Buckley CD. Inflammation across tissues: can shared cell biology help design smarter trials? Nat Rev Rheumatol. (2023) 19(10):666–74. doi: 10.1038/s41584-023-01007-2 37666996

[158] Ueda T Shiina S Iriguchi S Terakura S Kawai Y Kabai R. Optimization of the proliferation and persistency of CAR t cells derived from human induced pluripotent stem cells. Nat BioMed Eng. (2023) 7(1):24–37. doi: 10.1038/s41551-022-00969-0 36509913 PMC9870784

[159] Munoz AM Urak R Taus E Hsieh HJ Awuah D Vyas V. Dexamethasone potentiates chimeric antigen receptor t cell persistence and function by enhancing IL-7Ralpha expression. Mol Ther. (2024) 32(2):527–39. doi: 10.1016/j.ymthe.2023.12.017 PMC10861975 38140726

[160] Klysz DD Fowler C Malipatlolla M Stuani L Freitas KA Chen Y. Inosine induces stemness features in CAR-t cells and enhances potency. Cancer Cell. (2024) 42(2):266–82 e8. doi: 10.1016/j.ccell.2024.01.002 38278150 PMC10923096

[161] Ayala Ceja M Khericha M Harris CM Puig-Saus C Chen YY. CAR-t cell manufacturing: Major process parameters and next-generation strategies. J Exp Med. (2024), 221(2). doi: 10.1084/jem.20230903 PMC10791545 38226974

[162] Bashor CJ Hilton IB Bandukwala H Smith DM Veiseh O. Engineering the next generation of cell-based therapeutics. Nat Rev Drug Discovery. (2022) 21(9):655–75. doi: 10.1038/s41573-022-00476-6 PMC9149674 35637318

[163] McBride DA Jones RM Bottini N Shah NJ. The therapeutic potential of immunoengineering for systemic autoimmunity. Nat Rev Rheumatol. (2024). doi: 10.1038/s41584-024-01084-x 38383732

