# Supplementary material for: Immune mediated inflammatory diseases: moving from targeted biologic therapy, stem cell therapy to targeted cell therapy
Source: Front Immunol. 2025 Apr 7;16:1520063. doi: 10.3389/fimmu.2025.1520063 (PMC12009864; doi:10.3389/fimmu.2025.1520063)
Supplement: Supplementary file 1 [file DataSheet1.pdf]

Supplementary Materials

Immune mediated inflammatory diseases: moving from targeted biologic therapy, stem cell therapy to targeted cell therapy

Table S1. Clinical trials using CAR-T therapy for immune mediated inflammatory diseases

NCT Number	Study Title	Interventions
**Systemic Lupus Erythematosus**		
NCT05765006	CD19-CART(Relma-cel) for Moderate to Severe Active Systemic Lupus Erythematosus	BIOLOGICAL: Relma-cel
NCT05030779	A Study of CD19/BCMA Chimeric Antigen Receptor T Cells Therapy for Patients With Refractory Systemic Lupus Erythematosus	BIOLOGICAL: Assigned Interventions CD19/BCMA CAR T-cells
NCT05474885	BCMA-CD19 cCAR T Cell Treatment of Relapsed/Refractory Systemic Lupus Erythematosus (SLE)	BIOLOGICAL: BCMA-CD19 cCAR T cells
NCT05846347	Phase I Clinical Study of GC012F Injection in Treatment of Refractory Systemic Lupus Erythematosus	DRUG: GC012F injection
NCT05859997	Universal CAR-T Cells (BRL-301) in Relapse or Refractory Autoimmune Diseases	BIOLOGICAL: BRL-301
NCT05869955	A Study of CC-97540 in Participants With Severe, Refractory Systemic Lupus Erythematosus (SLE)	DRUG: CC-97540|DRUG: Fludarabine|DRUG: Cyclophosphamide
NCT05085418	A Study of CD19/BCMA Chimeric Antigen Receptor T Cells Therapy for Patients With Refractory Immune Nephritis	BIOLOGICAL: Assigned Interventions CD19/BCMA CAR T-cells
NCT05858684	Dual Target CAR-T Cell Treatment for Refractory Systemic Lupus Erythematosus (SLE) Patients	DRUG: GC012F injection
NCT03030976	A Study of CD19 Redirected Autologous T Cells for CD19 Positive Systemic Lupus Erythematosus (SLE)	DRUG: cyclophosphamide|DRUG: anti-CD19-CAR-T cells
NCT05938725	A Study of Anti-CD19 Chimeric Antigen Receptor T Cell Therapy for Subjects With Refractory Lupus Nephritis	Biological: KYV-101 anti-CD19 CAR-T cell therapy
NCT05798117	The study is intended to assess safety, efficacy and cellular kinetics of YTB323 treatment in participants with severe refractory systemic lupus erythematosus.	Single Group Assignment
NCT05930314	CNCT19 Cell Injection for Refractory Systemic Lupus Erythematosus	Refractory Systemic Lupus Erythematosus
NCT06222853	Study of Therapeutic Efficacy of Anti-CD19 CAR-T Cells in Children With Refractory Systemic Lupus Erythematosus	Biological: anti-CD19-CAR-T cells
NCT05988216	Universal CAR-T Cells (BRL-301) in Refractory Systemic Lupus Erythematosus	Biological: BRL-301
NCT06150651	Safety of PiggyBac Transposon CAR T-cells Targeting CD-19 in Refractory Lupus.	Other: CAR T-cell therapy

NCT06310811	Anti-CD19 CAR-T Cell Therapy in Participants With Moderate to Severe Active Systemic Lupus Erythematosus	Drug: RD06-04 Cells injection
NCT06106893	A Clinical Study of CD19 Universal CAR-γδT Cells in Refractory/Moderate-to-severe Systemic Lupus Erythematosus	Biological: CD19 Universal CAR-γδ T Cells
NCT06038474	Descartes-08 for Patients With Systemic Lupus Erythematosus (SLE-001)	
NCT06153095	A Study of IMPT-514 in Active Refractory Systemic Lupus Erythematosus (SLE)	Biological: IMPT-514
NCT06121297	RESET-SLE: A Phase 1/2 Open-Label Study to Evaluate the Safety and Efficacy of CABA-201 in Subjects With Active Systemic Lupus Erythematosus	Biological: CABA-201
NCT06294236	Study Evaluating SC291 in Subjects With Severe r/r B-cell Mediated Autoimmune Diseases (GLEAM)	Biological: SC291
**Sjogren's Syndrome**		
NCT05085431	A Study of CD19/BCMA Chimeric Antigen Receptor T Cells Therapy for Patients With Refractory Sjogren's Syndrome	BIOLOGICAL: Assigned Interventions CD19/BCMA CAR T-cells
**Scleroderma**		
NCT05085444	A Study of CD19/BCMA Chimeric Antigen Receptor T Cells Therapy for Patients With Refractory Scleroderma	BIOLOGICAL: Assigned Interventions CD19/BCMA CAR T-cells
NCT05869955	A Study of CC-97540, CD-19-Targeted Nex-T CAR T Cells, in Participants With Severe, Refractory Autoimmune Diseases	Drug: CC-97540/Drug: Fludarabine/Drug: Cyclophosphamide
NCT06152172	CARTIMMUNE: Study of Patients With Autoimmune Diseases Receiving KYV-101 (CARTIMMUNE)	Drug: KYV-101/Drug: Cyclophosphamide/Drug: Fludarabine
**Pemphigus Vulgaris**		
NCT04422912	Open-label Study to Determine the Maximum Tolerated Dose of DSG3-CAART in Mucosal-dominant PV Patients (mPV)	BIOLOGICAL: DSG3-CAART
**Neuromyelitis optica**		
NCT05828212	Evaluate the Safety and Efficacy of CAR-T Cells in the Treatment of R/R Neuromyelitis Optica	DRUG: CD19 CAR-T cells injection
NCT03605238	Treatment of Relapsed and/or Refractory AQP4-IgG Seropositive NMOSD by Tandem CAR T Cells Targeting CD19 and CD20	BIOLOGICAL: Corticosteroids & tanCART19/20
NCT04561557	Safety and Efficacy of CT103A Cells for Relapsed/Refractory Antibody-associated Idiopathic Inflammatory Diseases of the Nervous System	BIOLOGICAL: CT103A cells|DRUG: Cyclophosphamide and fludarabine
**Myasthenia Gravis**		
NCT05451212	Open-label Study to Evaluate the Safety of Various Dosing Regimens of MuSK-CAART for MuSK Myasthenia Gravis	BIOLOGICAL: MuSK-CAART

NCT05828225	Evaluate the Safety and Efficacy of CAR-T Cells in the Treatment of Refractory Myasthenia Gravis	DRUG: CD19 CAR-T cells injection
NCT04146051	Descartes-08 CAR-T Cells in Generalized Myasthenia Gravis (MG)	DRUG: Descartes-08
NCT04561557	Safety and Efficacy of CT103A Cells for Relapsed/Refractory Antibody-associated Idiopathic Inflammatory Diseases of the Nervous System	BIOLOGICAL: CT103A cells|DRUG: Cyclophosphamide and fludarabine
NCT06193889	A Study of Anti-CD19 Chimeric Antigen Receptor T-Cell Therapy, in Subjects With Refractory Generalized Myasthenia Gravis	Biological: KYV-101/Drug: Standard lymphodepletion regimen
**Autoimmune Diseases**		
NCT05239702	Clinical Study of Targeting CD7 CAR-T Cells in the Treatment of Autoimmune Diseases	BIOLOGICAL: CD7 CAR T-cells
NCT05263817	A Clinical Study of CD19/BCMA CAR-T Cells in the Treatment of Refractory POEMS Syndrome, Amyloidosis, Autoimmune Hemolytic Anemia, and Vasculitis	BIOLOGICAL: CD19/BCMA CAR T-cells
NCT05459870	CAR-T Cells Targeting B Cell Related Autoimmune Diseases	Single Group Assignment
NCT06279923	CD19-BAFF CAR-T Cells Therapy for Patients With Autoimmune Diseases	Biological: CD19-BAFF Targeted CAR T-cells
NCT06056921	Safety and Efficacy of CD19 Targeted CAR-T Therapy for Refractory Autoimmune Disease	Biological: CD19 targeted CAR-T cells
**Inflammatory Diseases of the Nervous System**		
NCT04561557	An Open Label Clinical Trial to Evaluate the Safety and Efficacy of CT103A Cells for the Treatment of Relapsed/Refractory Antibody-associated Idiopathic Inflammatory Diseases of the Nervous System	Sequential Assignment

Table S2. Recent meeting abstract using CAR-T therapy for immune mediated inflammatory diseases

First author	Year	Disease	Recognition domain	Carrier cells	Target cells	Clinical study	Dosage	Persistence
**Abstract (ACR 2023)**								
Christina Bergmann	2023	Systemic sclerosis	CD19 scFv	CAR T	CD19+B cells	In vivo		
Josefina Cortés Hernández	2023	SLE	CD19 scFv	CAR T	CD19+B cells	In vivo	12.5*10^6	28 days
Georg Schett	2023	SLE	CD19 scFv	CAR T	CD19+B cells	In vivo	1.0*10^6/kg	14 months
Jérôme Avouac	2023	Systemic sclerosis	CD19 scFv	CAR T	CD20+CD19+B cells	In vivo	20*10^6	6 weeks
Jule Taubmann	2023	Refractory Antisynthetase Syndrome	CD19 scFv	CAR T	CD19+B cells	In vivo		
Yong Yuan	2023		CD19 scFv	cCAR	BCMA-CD19+B cells	In vivo	1.5-3x10^6/kg	6 months
**Abstract (EULAR 2024)**								
Y. Ma.	2024	SLE		cCAR		In vivo	3*10^6/kg	150 days
J. Taubmann	2024	Refractory Antisynthetase Syndrome	CD19 scFv	CAR T	CD19+B cells	In vivo	1.0*10^6 CAR T cells/kg	18 months
J. Auth	2024	Systemic sclerosis	CD19 scFv	CAR T	CD19+B cells	In vivo	1.0*10^6 CAR T cells/kg	3-4 months
G. Schett	2024	Rheumatic disease	CD19 scFv	CAR T	CD19+B cells	In vivo	1.0*10^6/kg	6 weeks
J. Cortés-Hernández	2024	SLE	CD19 scFv	CAR T	CD19+B cells	In vivo	12.5*10^6	
L. Bucc	2024	Autoimmune disease	CD19 scFv	CAR T	CD19+B cells	In vivo		4 months
R. Nicolai	2024	Rheumatic disease	CD19 scFv	CAR T	CD19+B cells	In vivo		
S. Albani	2024	Autoimmune disease		CAR Treg		in vitro/In vivo		
A. Mahne	2024	Autoimmune disease	CD19 scFv	CAR T	CD19+B cells	In vivo		

J. Chou	2024	Autoimmune disease	CD19 scFv	CAR T	CD19+B cells	In vivo		
J. Henes	2024	Systemic sclerosis	CD19 scFv	CAR T	CD19+B cells	In vivo		
C. Bracaglia	2024	SLE	CD19 scFv	CAR T	CD19+B cells	In vivo	1.0*10^6/kg	3 months
**Abstract (EHA 2024)**								
M Doglio	2024	SLE	CD19 scFv	CAR Treg	CD19+B cells	in vitro/In vivo	3.5 × 10^6	8 weeks
